# Supplementary figures and images for: Protein-Protein interactions uncover candidate ‘core genes’ within omnigenic disease networks
Source: PLoS Genet. 2020 Jul 17;16(7):e1008903. doi: 10.1371/journal.pgen.1008903 (PMC7390454; doi:10.1371/journal.pgen.1008903)

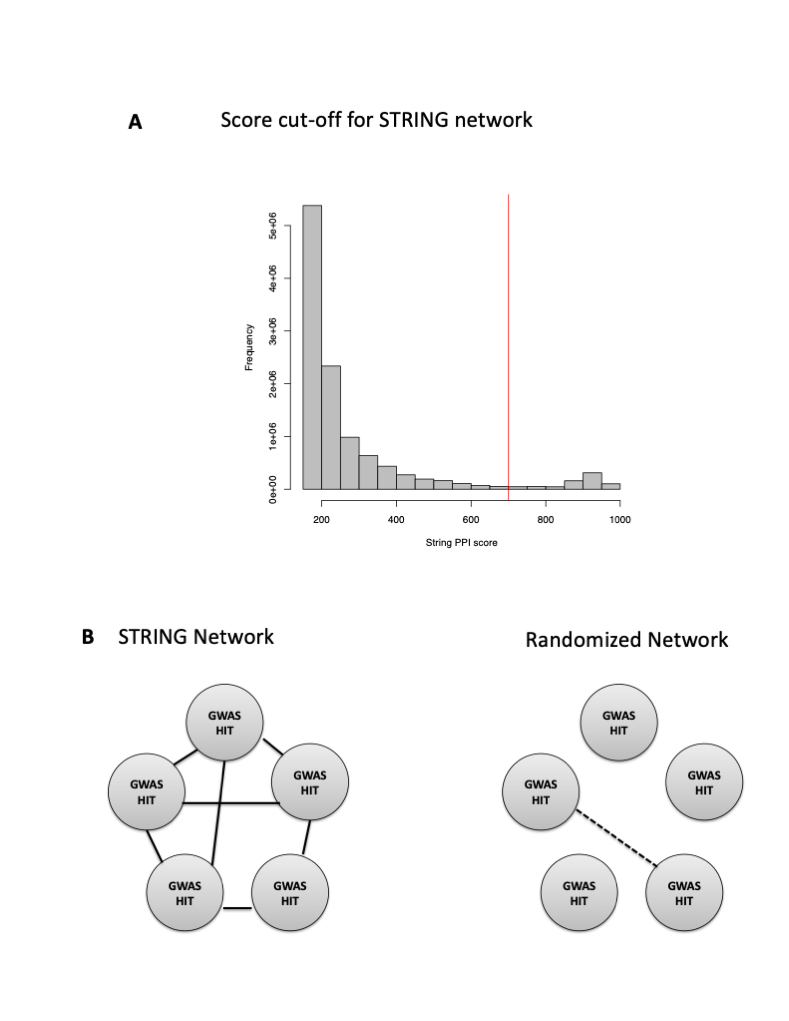

Supplement: S1 Fig — (A) This figure shows the distribution of scores within the STRING PPI network. The scores ranged from 150–999, with a mean of 277.65. We restricted our analyses to PPI interactions with score > = 700, representing 6% of all PP interactions in the network. The red vertical line represents the score cut-off of 700 we chose for our analysis. (B) Schematic showing that the observed number of PPI among GWAS hits in the STRING network tends to be higher than the number of PPI among GWAS hits in randomized networks. (TIF) [file pgen.1008903.s001.tif]

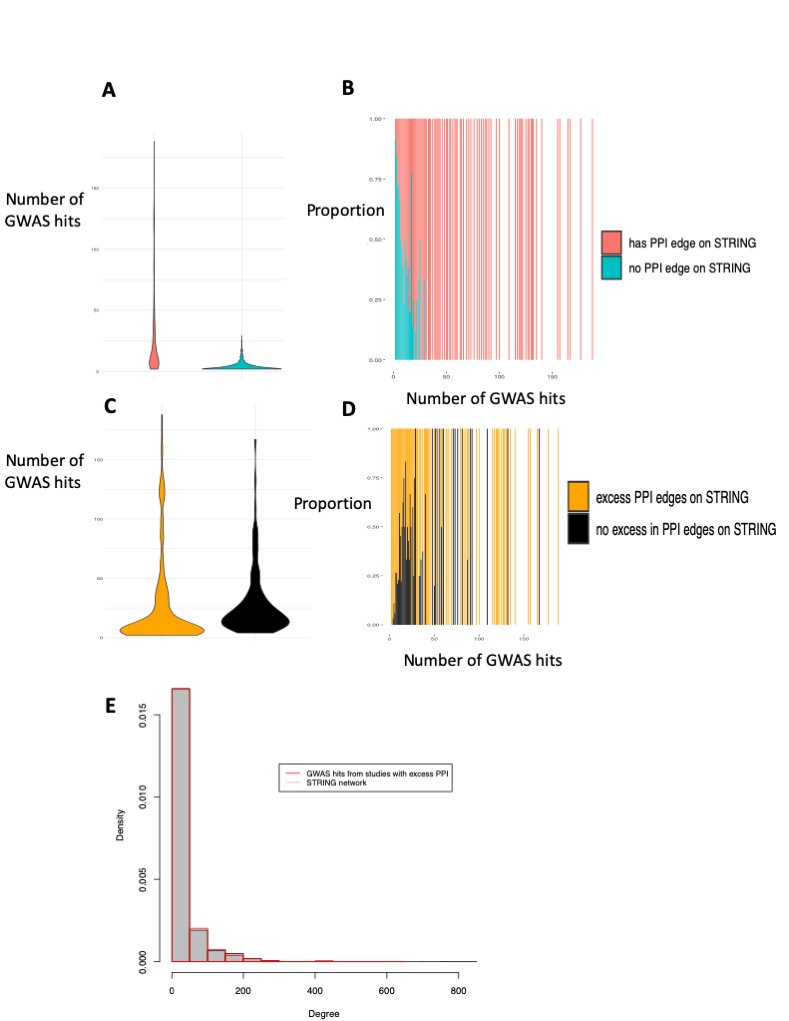

Supplement: S2 Fig — (A) Distribution of GWAS hits amongst studies with at least 1 PPI edge on STRING, and studies with no PPI edge between GWAS hits. (B) The proportion of studies that have at least 1 PPI edge at each number of GWAS hits. This plot demonstrates that at low GWAS hits the likelihood of having at least 1 PPI edge between the GWAS hits is low, but as the number of GWAS hits increases the chances of having at least 1 PPI edge between the GWAS hits increases. (C) Distribution of GWAS hits amongst studies with excess PPI edges between GWAS hits and amongst studies with no excess in PPI edges between GWAS hits. (D) The proportion of studies with excess PPI edges at each number of GWAS hits. Although there is a tendency for studies with low GWAS hits to have no excess in PPI edges, there are many studies with high numbers of GWAS hits that do not have excess PPI edges. (E) In gray is the degree distribution of the whole STRING network, and in red is the degree distribution of GWAS hits within GWAS studies with excess PPI (n = 270). (TIF) [file pgen.1008903.s002.tif]

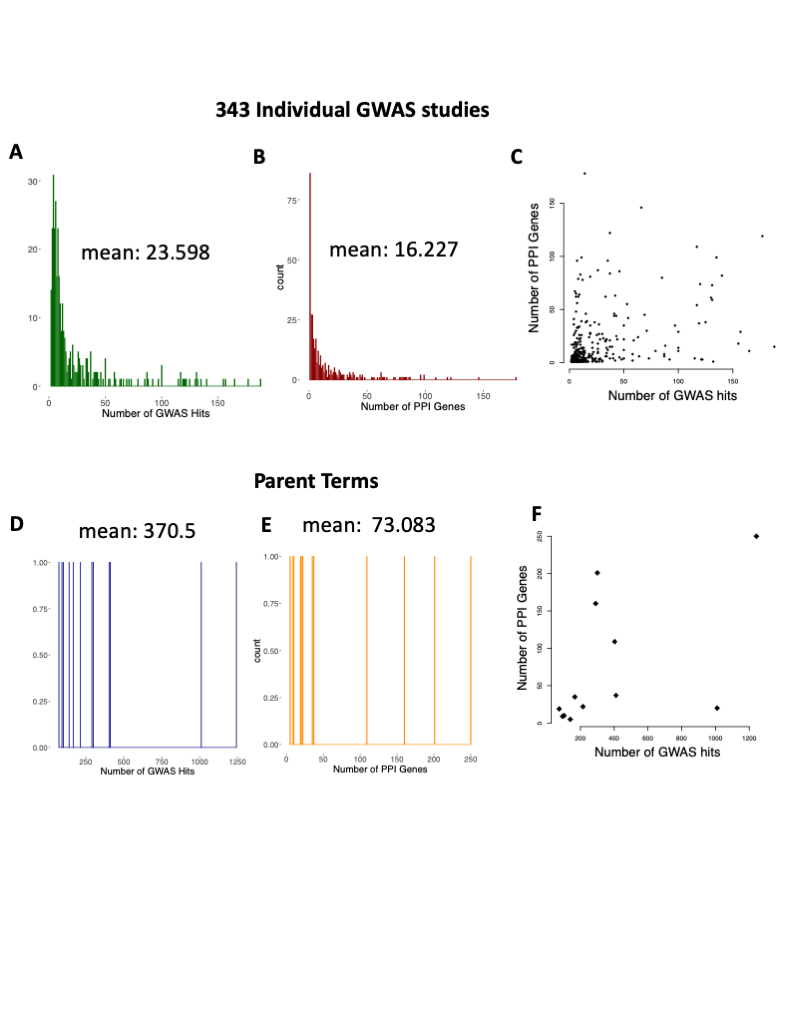

Supplement: S3 Fig — (A) Distribution of number GWAS hits in the 343 Individual GWAS studies we detected PPI genes in. (B) Distribution of PPI genes in the 343 individual GWAS studies we detected PPI genes in. (C) Scatter plot of number of GWAS hits in each study compared to the number of PPI genes in each study, showing very little correlation. (D) Distribution of the number of GWAS hits in 12 parent terms that we detect PPI genes in. (E) Distribution of PPI genes in the 12 parent terms that we detect PPI genes in. (F) Scatter plot of the number of GWAS hits in each parent term compared to the number of GWAS hits. (TIF) [file pgen.1008903.s003.tif]

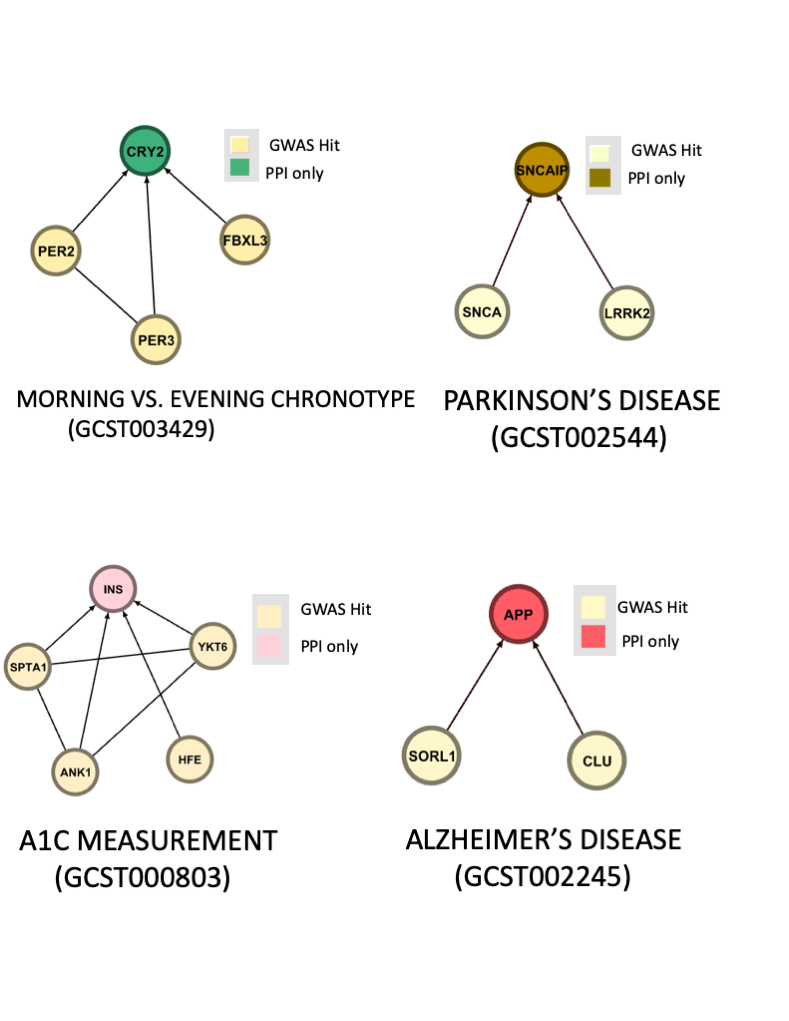

Supplement: S4 Fig — PPI genes in Morning vs. Evening chronotype, Parkinson’s Disease, A1C Measurement and Alzheimer’s Disease. The GWAS catalog study accessions are shown within parentheses. (TIF) [file pgen.1008903.s004.tif]

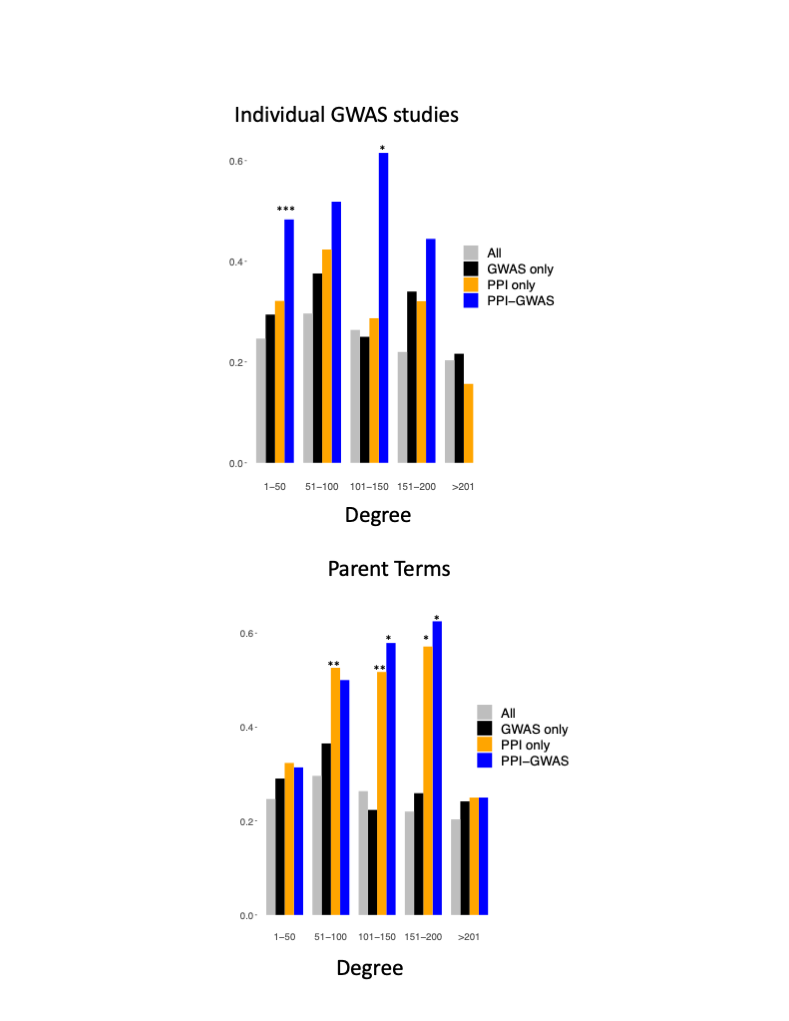

Supplement: S5 Fig — Individual GWAS studies: after stratifying by degree-bin we found that PPI-GWAS had the highest proportion of Clinvar pathogenic variants across 4 degree bins (statistically significant in 2 bins) consistent with what was observed prior to stratifying by degree. Parent Term analysis: we found that PPI only had a greater proportion than GWAS only in 3 bins, and PPI-GWAS has a greater proportion than GWAS only in 2 bins. (TIF) [file pgen.1008903.s005.tif]

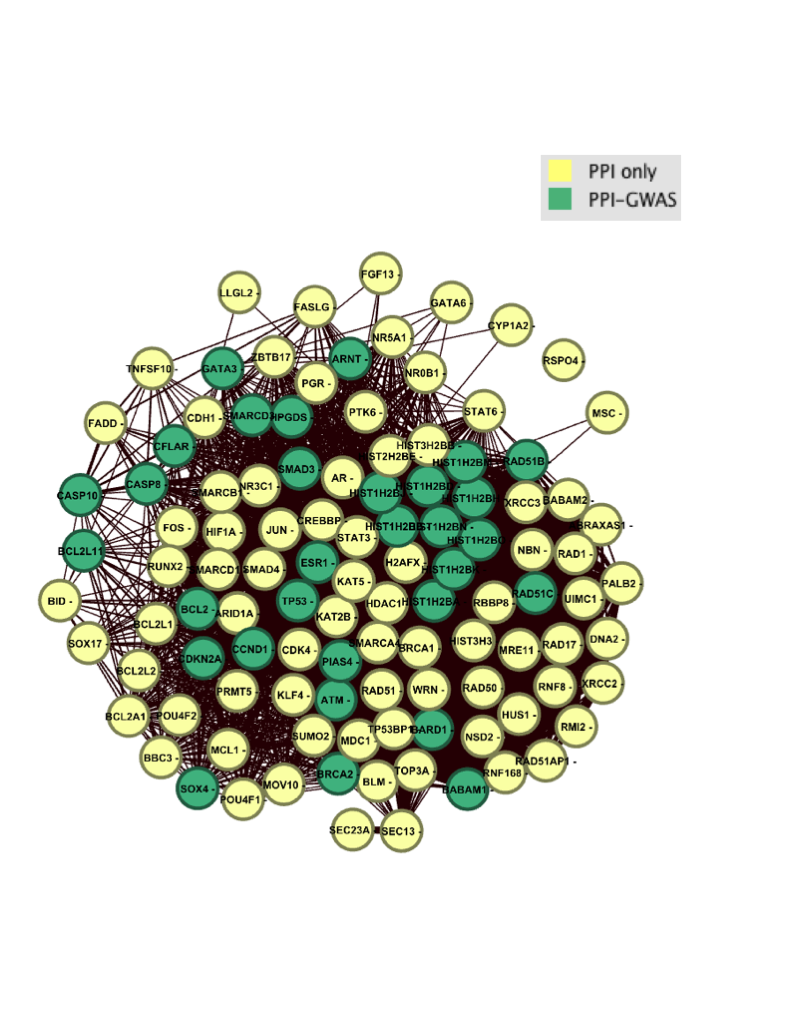

Supplement: S6 Fig — PPI only are shown in yellow while PPI-GWAS are shown in green, and PPI edges are represented as lines. We used the STRING network to extract the directly interacting partners of each of the 109 cancer parent term PPI genes. Then we did a pairwise comparison to count the number of directly interacting partners that are shared across each node pair, and then we used the number of shared interacting partners to weight the edge between the 2 nodes. We then used the Fruchterman Reingold force directed layout algorithm [60] within Gephi [59] to visualize the network. (TIF) [file pgen.1008903.s006.tif]

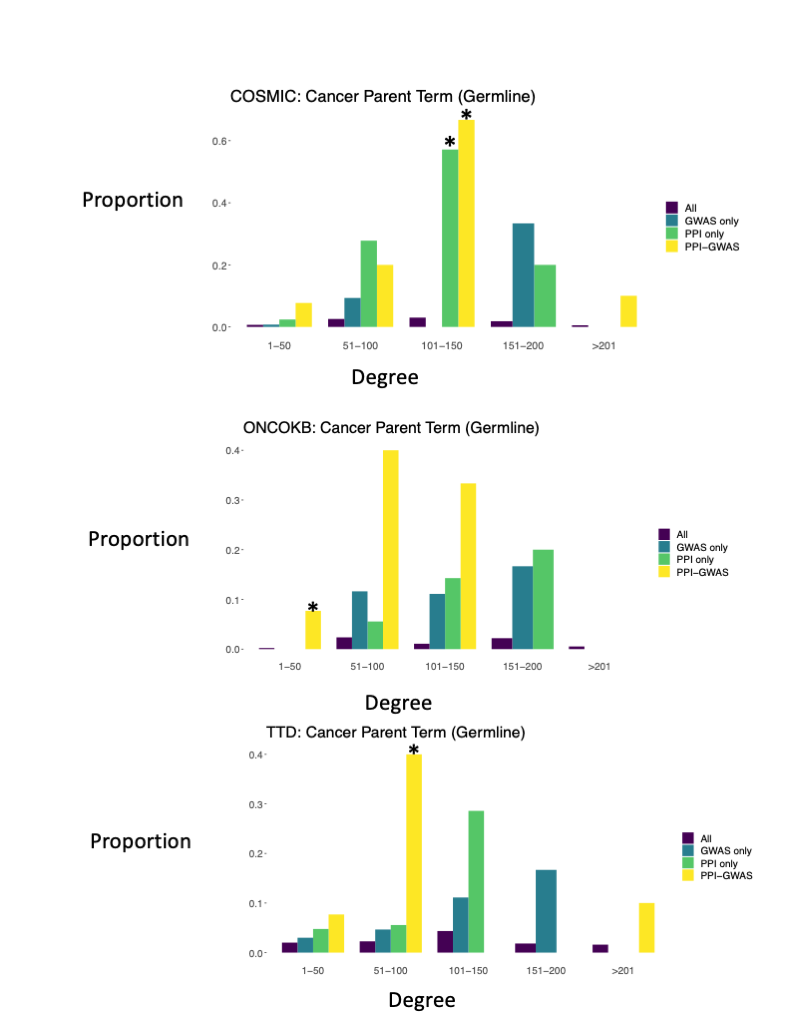

Supplement: S7 Fig — Similar to the unstratified analysis we found that PPI-GWAS and PPI only were enriched for COSMIC germline variants, and PPI-GWAS was enriched compared to GWAS only in Oncokb drug targets and PPI-GWAS was enriched compared to GWAS only in Therapeutic Targets Database cancer drug targets. (TIF) [file pgen.1008903.s007.tif]

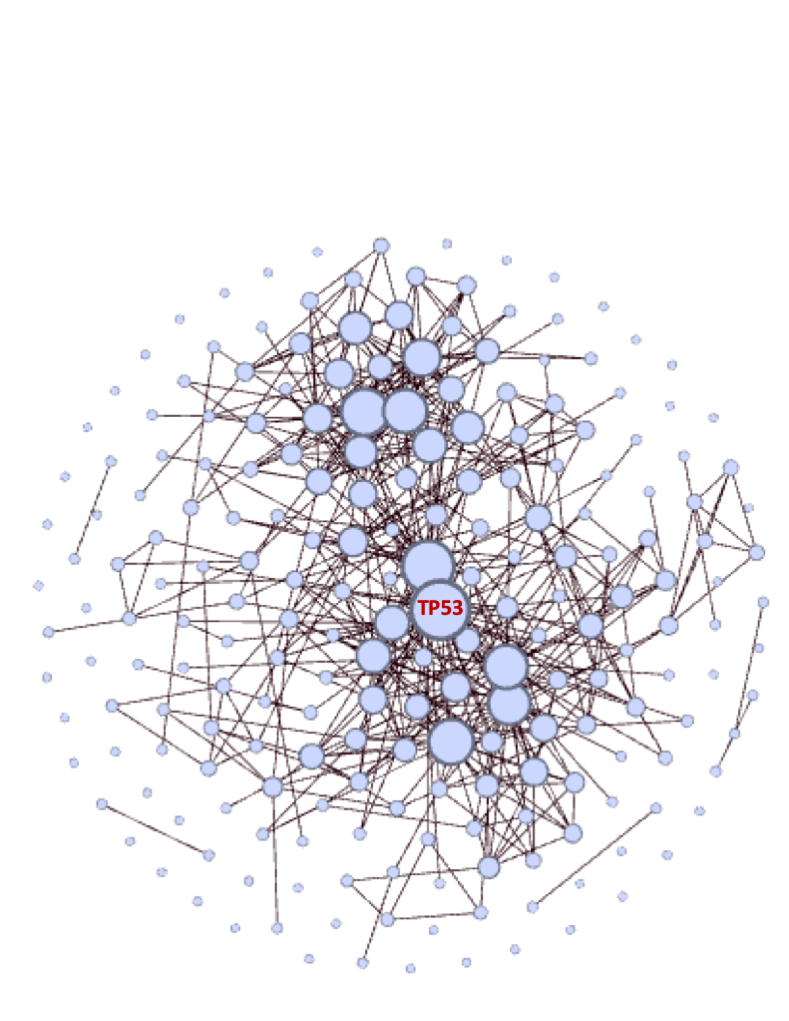

Supplement: S8 Fig — The size of the node corresponds to the degree, and PPI are represented with lines. (TIF) [file pgen.1008903.s008.tif]

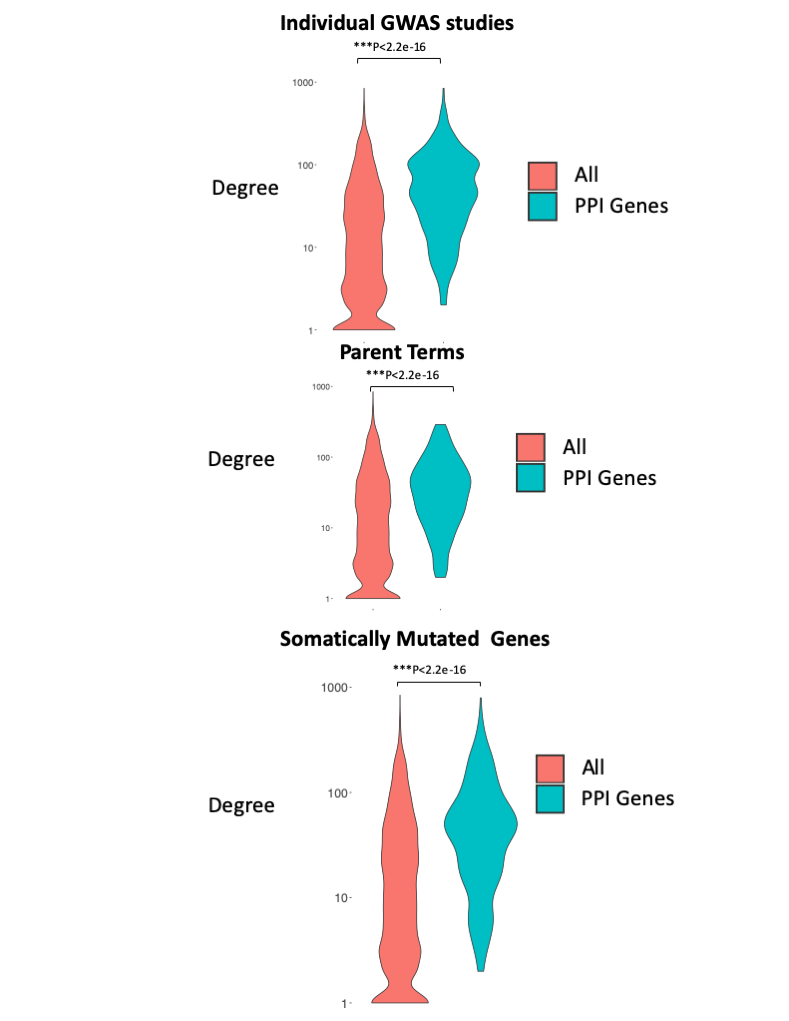

Supplement: S9 Fig — (TIFF) [file pgen.1008903.s009.tiff]

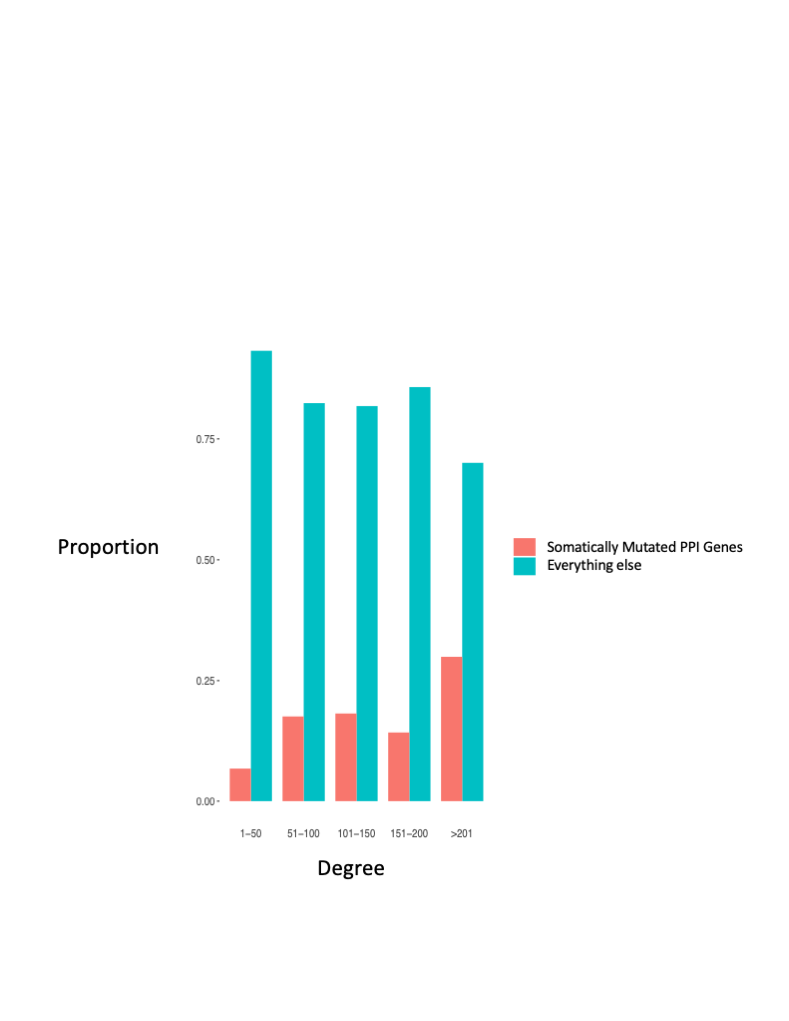

Supplement: S10 Fig — (TIF) [file pgen.1008903.s010.tif]

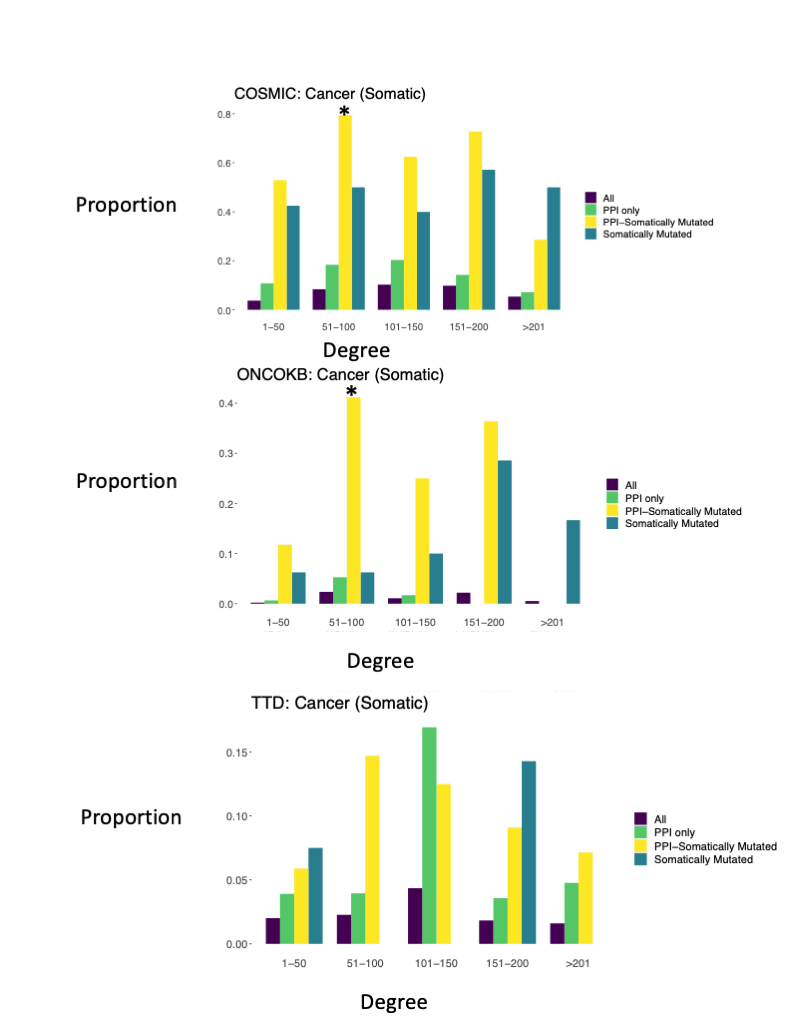

Supplement: S11 Fig — Similar to the unstratified analysis we found that compared to Somatically Mutated, PPI-Somatically mutated was enriched for COSMIC somatic variants, and PPI-Somatically Mutated was enriched compared to Somatically Mutated in Oncokb drug targets. (TIF) [file pgen.1008903.s011.tif]
