# Supplementary material for: Protein-Protein interactions uncover candidate ‘core genes’ within omnigenic disease networks
Source: PLoS Genet. 2020 Jul 17;16(7):e1008903. doi: 10.1371/journal.pgen.1008903 (PMC7390454; doi:10.1371/journal.pgen.1008903)
Supplement: Attachment — Submitted filename: Response_to_reviewers.pdf [file pgen.1008903.s030.pdf]

Editor's comments

Although the reviewers commented favorably on the general goal and approach of the manuscript, there were significant criticisms that preclude publications. These comments indicate that significant amount of re-analysis is need to make the paper convincing (See comments of reviewer1 and reviewer2). **"Core genes" or the gene targets that your approach aims to identify need to be carefully defined,** and additional analyses need to support your claims on the findings. We would be willing to reconsider the manuscript after it has undergone a major revision that takes into account of the criticisms of the reviewers, with no assurance of acceptance

We thank the reviewers and editor for their careful reading of our manuscript and their constructive criticisms which we believe have helped strengthen the work. We have now addressed the points the reviewers brought up, and provide point-by-point responses below. Due to the number of technical terms being defined, we have now included a definitions box (**lines 209-219**) that includes terms commonly used in the manuscript, and have included a definition of core genes in the introduction (line 87-88): ***"'core genes' which have direct effects on pathways central to pathogenesis (e.g. synaptic genes in schizophrenia(Boyle et al. 2017))"***

Reviewer 1

Reviewer #1: In this paper, the authors present a method to identify genes which are enriched for protein-protein interactions (ppi) with genes near GWAS loci (or some other list of genes). The authors argue that these represent "core genes" under the omnigenic model. They describe some examples where the identified genes are known to be highly disease-relevant, and they report that their list of genes is enriched for signal according to Clinvar and according to pLI.

I tend to think the authors are onto something with this method, but more work is needed before the paper is convincing. **In particular, the authors must distinguish between the following claims: (1) genes with high ppi to GWAS-proximal genes, but without GWAS hits themselves (ppi-only genes), are bonafide disease-relevant genes. (2) among GWAS-proximal genes, those with ppi to other GWAS-proximal genes (ppi-gwas genes) are more disease relevant than those without (gwas-only genes). (3) ppi-only genes, in addition to being disease relevant, are even more disease relevant than gwas-only genes.**

We thank the reviewer for noting this important distinction between three categories of genes that are important for understanding the analysis we present. In the analysis of individual GWAS studies and parent terms, we have followed the reviewer's suggestion and clearly separated analysis for these three categories using the reviewers suggested nomenclature, namely 'PPI only', 'PPI-GWAS' and 'GWAS only'. In the somatically mutated cancer genes analysis we have used the categories 'PPI only', 'PPI-Somatically Mutated' and 'Somatically Mutated'.

This is the sequence of claims that is necessary to justify calling the ppi genes "core", implying that they are more important than most GWAS genes, which are presumably peripheral. The authors have provided evidence for (1), but not for (2-3). I have some suggestions for how they could make these comparisons. However, I do not think that acceptance of the paper should be contingent on identifying strong evidence for both these claims (see below).

The ClinVar and pLI comparisons are made between ppi genes (including both ppi-gwas and ppi-only) and all genes. There is also a comparison with "parent" genes, i.e. genes with high ppi to the larger set of parent-term GWAS-proximal hits. Missing is a comparison with gwas-only genes. **I would like to see a comparison between ppi-gwas and gwas-only, and between ppi -only and gwas-only.** These comparisons could provide some evidence of (2) and (3) respectively.

We thank the reviewer for these suggestions and have now performed these analysis for both the Clinvar and pLI comparisons. As anticipated, we found a stronger enrichment for Clinvar pathogenic variants in PPI-only compared to GWAS only and PPI-GWAS compared to GWAS only in both the individual GWAS studies, and the parent term analysis.

We have subsequently updated **Figure 3c and 4c** as below.

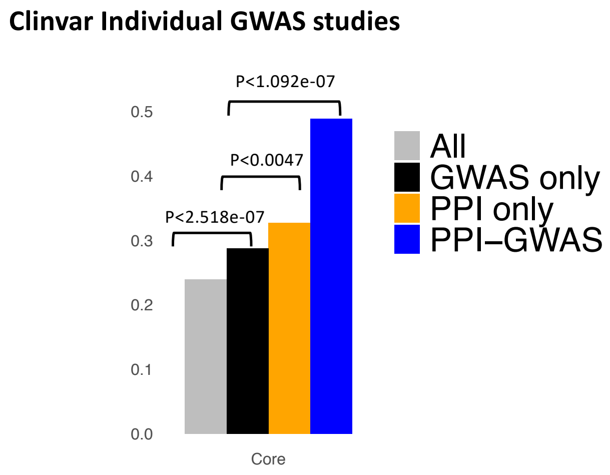

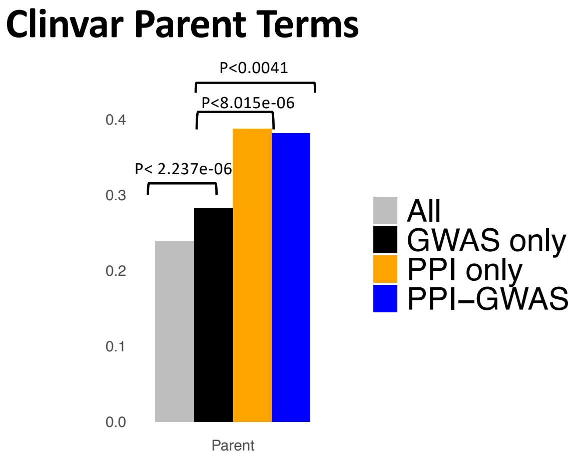

We also updated the description of the results to include the lines below:

Lines 306-312

*We looked for overlaps between our candidate core genes and genes with pathogenic variants within Clinvar(Landrum et al. 2016) (**Figure 3c, S13 Table**), and found a higher proportion of Clinvar pathogenic variants in PPI only compared to GWAS only (33% vs. 29% respectively, p<0.0047, **Figure 3c**) and PPI-GWAS compared to GWAS only (49%*

vs. 29%, p<1.092e-07) . Interestingly we also found GWAS only was enriched compared to the whole network (29% vs. 24%, p< 2.518e-07)

Lines 399-409

*Lastly, we explored overlap between parent term candidate core genes and genes containing pathogenic variants within Clinvar(Landrum et al. 2016) (**Figure 4c**). We found a higher proportion of Clinvar pathogenic variants in PPI only compared to GWAS only (39% vs. 28%, p<8.015e-06) (**Figure 4c**), and between PPI-GWAS and GWAS only (38% vs. 28%, p<0.004) and GWAS only and the whole network (28% vs. 24%, p< 2.237e-06). Interestingly, the PPI only candidate core genes from the parent term analysis identified a stronger association with Clinvar than individual studies (39% vs. 33%, p<0.015).*

However, performing the pLI analysis this way doesn't correct for degree node. So we repeated the analysis after binning the nodes by degree ranging from (1-50, 50-100, 101-150, 150-200, >201), and after binning we did not find any strong enrichment in pLi across the candidate core genes (see below), and as a result have removed this analysis from the manuscript.

pLI Individual GWAS studies

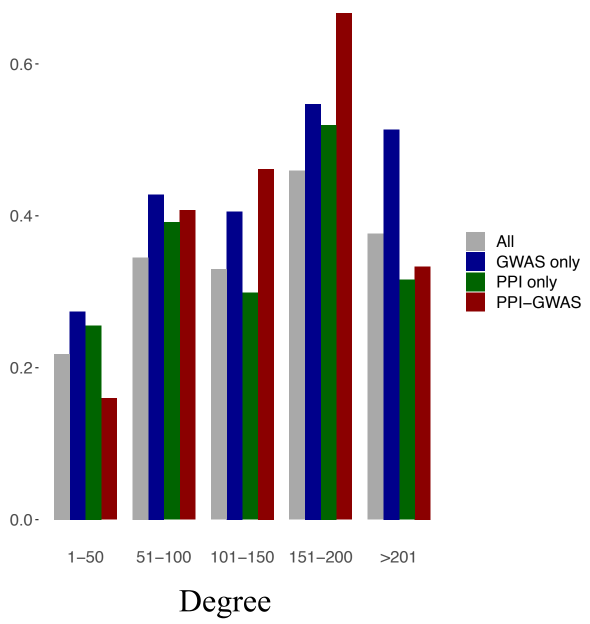

pLI Parent Terms

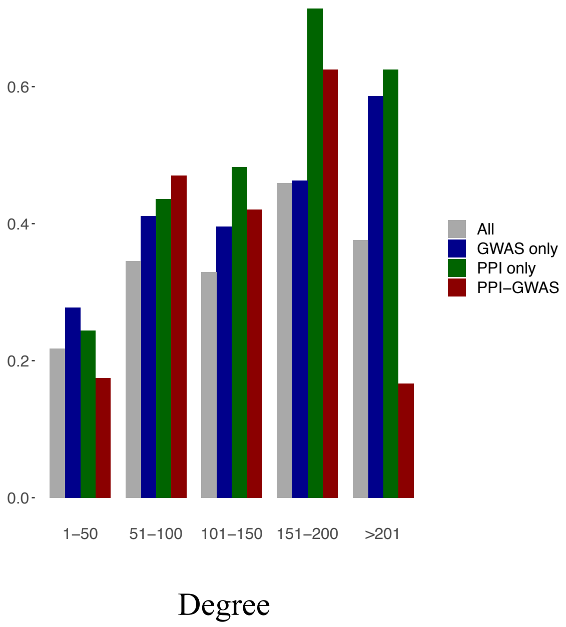

Moreover, are ppi-only genes enriched for heritability, even if they do not harbor significant GWAS hits?

We thank the reviewer for this interesting suggestion, and agree that enriched heritability could provide evidence for the 'importance' of candidate core genes. However, we were unsure how to do this. We downloaded per SNP heritability estimates from the UK biobank (https://nealelab.github.io/UKBB_ldsc/) for phenotypes similar to the GWAS phenotypes, and then extracted per SNP heritability estimates from common variants (MAF>0.05) that overlap the genes in GWAS only, PPI only, PPI-GWAS, all 11,049 genes in our network and all common SNPs. Then for each list of genes we summed across the absolute value of the per SNP heritability values, then divided by the total number of SNPs in that category (see table below). In the 'Lipid and Lipoprotein Measurement' parent term, we found that the SNPs in PPI-GWAS had higher per-SNP heritability than the SNPS in GWAS only. However, we found little evidence that PPI only genes are enriched for heritability compared to other genes in the genome. Below we show results for selected parent terms.

Parent term	UK biobank phenotype	GWAS only	PPI only	PPI-GWAS	11409 genes	All SNPs
Lipid_or_lipoprotein_measurement	Cholesterol	2.860	0.912	5.012	1.029	0.993
Cancer	Breast Cancer	0.973	0.890	1.312	0.833	0.828
Cancer	Prostate Cancer	0.989	0.822	0.900	0.837	0.839
Hematological_measurement	Mean platelet volume	2.458	1.462	2.438	1.192	1.203
Digestive_system_disorder	Diseases of the digestive system	0.955	0.955	0.999	0.891	0.886
Cardiovascular_disease	Major coronary heart disease event	1.065	0.903	0.703	0.859	0.849

Are ppi-gwas genes more likely to be among the very most significant genes, compared with gwas-only genes

We thank the reviewer for this interesting suggestion. We only performed this analysis on the individual GWAS studies, because the parent term analysis involved aggregating GWAS hits (and thus p-values) across many GWAS studies. We identified candidate core genes in 343 individual GWAS studies, and of these, only 99 GWAS studies contained at least 1 PPI-GWAS. Across the 99 GWAS studies there were 4,900 GWAS hits in total, and out of these 313 were PPI-GWAS. We extracted from the GWAS catalog the GWAS p-values for all of the GWAS hits in each of these 99 studies, and then ranked all of the GWAS hits in each study by their GWAS p-value (more significant p-values had lower ranks).In order to facilitate comparison across GWAS studies we divided this rank by the number of GWAS hits in each study to calculate the rank fraction.

Rank fraction = p-value rank in GWAS study/number of GWAS hits in study.

We then plotted the distribution of rank fractions across all GWAS hits (in gray in **Figure 3d**, also included below **a.**). The distribution is uniform, as would be expected, and then in red we plot the distribution of rank fractions for only the PPI-GWAS (red, in **Figure 3d**, also included below in **a.**). We found that the distribution of rank fractions from PPI-GWAS did not follow a uniform distribution, but were skewed to the left, indicating that PPI-GWAS are more likely to be among the most significant GWAS hits, in each study. To better visualize this, we plotted the rank of each GWAS hit in each study in black, highlighting the PPI-GWAS in red(**Figure 3e**, also included below **b.**), and found that PPI-GWAS tend to cluster at lower ranks (towards the left).

Lastly, we used a Fisher's Exact Test to quantify this enrichment. We found that 42% of PPI-GWAS were ranked in the top 25% of P-values ($p < 1.638e-11$) while 17% were ranked in the top 10% ($p < 2.333e-05$). We have used this finding as evidence that PPI-GWAS are more disease relevant than GWAS only, and have included this in the results section (**lines 321-326**) and discussion section (**lines 578-581**) and have included the data in (**S3 Table**).

a.

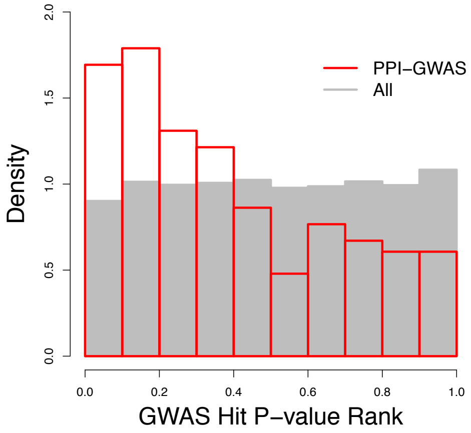

b.

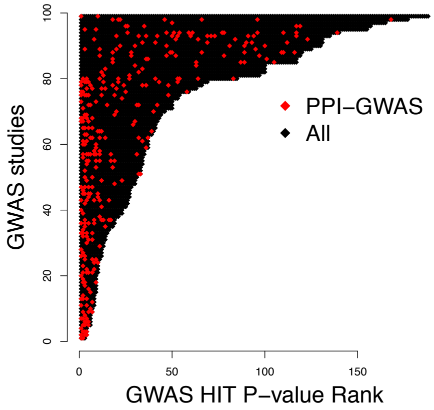

For each parent category, is the ClinVar enrichment signal concentrated among genes associated with parent-category-related pathologies?

We compared parent term candidate core genes that contain Clinvar pathogenic variants to all genes in the network (n=11,049) and used MSigdb (Liberzon et al. 2015) to identify any enriched pathways, and found evidence for enrichment in disease relevant pathways across a number of parent terms and have included representative examples in **S6 Table**. For instance in the parent term Cardiovascular Disease the top 2 most enriched pathways were "***Kegg complement and Coagulation Cascades***", and "***Reactome Formation of Fibrin clot clotting cascade***" which were driven by the parent term candidate core genes *F2, SERPIND1, F10, F9, F11, SERPINF2, PROC, CFD, THBD* and *F2, F9, F10, F11, PROC, THBD* respectively.

In "Lipid or Lipoprotein Measurement" we found the 6th most enriched pathway "***GO Protein Lipid Complex***", which was driven by the candidate core genes *APOB, APOA4, APOA1, APOC3, LPL, APOE, VLDLR.* In Cardiovascular measurement we found enrichment in the pathway ***"GO Regulation of Heart Contraction"*** which was

driven by the candidate core genes *GATA4, KCNA5, KCND3, SCN5A, KCNQ1, KCNH2, ATP2A1*. In the Cancer parent term we found enrichment in ***"GO DNA synthesis involved in DNA repair***" which was driven by the candidate core genes *PALB2, RBBP8, BLM, BRCA1, TOP3A, RAD51, BRCA2, RAD51C, ATM, WRN, NBN, DNA2, BARD1, XRCC2, RAD50.* We used these results to demonstrate that the Clinvar enrichment signal in parent term candidate core genes is driven by disease relevant genes in the results section (**lines 406-409**).

Even if all these analyses are positive, a critical caveat is that many GWAS-proximal genes will not actually be the right gene. **(Could it be possible to observe this empirically, by showing that the 2nd closest gene to each GWAS hit is also enriched with ppi to other GWAS-proximal genes and this cannot be explained by proximal genes having shared ppi?)**

We agree with the reviewer that a limitation of this work is the assumption that the closest gene to the GWAS hit, is the causal gene, and agree that the assigned gene may be incorrect. Following the reviewer's suggestion, we sought to evaluate the proportion of studies that have excess PPI if we replace the GWAS hits with the second closest gene. We performed 2 different analyses.

In the first scenario we only replaced 1 of the GWAS hits with the second gene, when we did this, we found that 315 studies had at least 1 PPI edge and of these, 158 were enriched for PPI. In the second scenario, we replaced all of the GWAS hits with the second closest gene and found that 268 studies had least 1 PPI edge and out of these only 56 were enriched for PPI.

Of note, in our original analysis we found that 365 studies had at least 1 PPI edge on the STRING network, and of these 270 studies had excess PPI among GWAS hits (P<0.05).When we compare the proportion of studies with excess PPI, we found that it decreases as we replace more of the GWAS hits with he second closest gene.

270/365 > 158/315 > 56/268
74% > 50% > 21%

However, the fact that we still see GWAS studies with excess PPI after replacing all the GWAS hits, suggests that the second closest gene is likely to be the causal gene in at least some of the studies. We described this analysis in the results section (**lines 159-167**) and the discussion section (**lines 627-629**).

The reason this caveat can affect these analyses is that it is difficult to distinguish between two possibilities: (1) ppi-gwas genes are more likely to have ClinVar phenotypes because they are more important for disease than gwas-only genes, even though the gwas-only genes are also bona fide disease genes; and (2) ppi-gwas genes are more likely to have ClinVar phenotypes because they are more likely to be the true disease gene, whereas gwas-only genes are more likely to be the wrong gene at the

locus altogether (but the correct gene, if we knew it, would be just as important as a ppi-gwas gene). I think the authors need to discuss this distinction. I don't see a way that they can realistically rule out possibility 2, but that's ok: their list of genes is interesting under either possibility 1 or possibility 2.

We agree with the reviewer that one possible interpretation is that GWAS only genes perhaps have the wrong gene at the locus, and have now added this to the limitations section in the discussion (**lines 631-638**). Although it is difficult to distinguish between these two scenarios, in our Clinvar analysis we found that compared to the whole network, GWAS only genes were also enriched for Clinvar pathogenic variants. Further, the fact that we see a greater proportion of studies with excess PPI when we do not replace with the second closest gene suggests that the GWAS only genes in our study are more likely to be causal compared to the 2nd closest gene. Suggesting that the GWAS only genes are more likely to be the true disease gene at least some of the time. That being said, we agree with the reviewer that it is not fully possible to disentangle these two possibilities with the data-at-hand although, overall we feel that possibility 2 plays less clear role based on our interpretation of the totality of the data.

If the suggested analyses are negative, I don't think it should necessarily preclude publication of this paper, but I would think that some re-writing would be needed. At a minimum, "core genes" should be replaced with "GWAS-interacting gene" or some other, less aspirational term. It's still fine to motivate the network analyses using the omnigenic model, and say that your goal is to identify core genes, but if there is evidence that the identified genes are any more "core" than other GWAS genes, then it doesn't seem appropriate to call them "core genes". You can call p53 a core gene in cancer, but it doesn't follow that all the other ppi genes are just as important.

I want to emphasize again that it is still valuable to identify new disease-relevant genes via ppi with other disease genes, even if you aren't claiming that they are all "core" or even enriched for core genes relative to other GWAS genes.

Although the pLI and heritability enrichment analyses were negative, all the other analyses were positive, so we believe we are detecting core genes, however in the manuscript we refer to them as 'candidate core gene' and in the discussion section we argue that they are 'core genes'(**lines 575-588**).

I have some minor comments as well:
1. I noticed that many HLA genes are detected, and usually they are annotated as "new" even for phenotypes with clear HLA associations. I think there may be some issue at HLA where only one gene is being picked as the GWAS gene, and the others are being called "new" even though this obviously isn't appropriate at this locus. Probably change the "new" criterion to "not within x Kb of the lead SNP", and possibly exclude HLA from all analyses.

Thank you for this suggestion. Instead of directly removing HLA loci, we decided to create two additional filters. The first filter re-categorizes candidate core genes within

1Mb of the lead SNP from ‘PPI only’ to ‘PPI-GWAS’. The second filter removes instances where all of the GWAS hits that detect a candidate core gene come from the same locus, by requiring that each candidate core gene is detected by at least 2 GWAS hits that are not within 1MB of each other.We applied both of these filters to the candidate core genes detected from individual GWAS studies, parent terms and somatically mutated cancer genes, and have included a description of how this was done in the methods section (**lines 816-826**). We noticed that after applying the second filter many of the HLA candidate core genes disappeared.

2. The omnigenic model is usually described in terms of gene-regulatory networks, not ppi networks. I don’t think this distinction is critical to the model, but maybe the authors could clarify that ppi is only one way that genes can interact, that it is not the type of interaction most people would associate with the omnigenic model, but that it is just as plausible for peripheral gene effects to be mediated by ppi as it is for them to be mediated by gene-regulatory networks.

We thank the reviewer for this suggestion and have now added the following lines to the discussion (**lines 606-609**):

“Although the Omnigenic model is usually described in terms of gene-regulatory networks(Boyle et al. 2017), it is just as plausible for peripheral gene effects to be mediated by PPI as it is for them to be mediated by gene-regulatory networks”

In addition, the authors could either apply their method to some gene-regulatory networks or explain why they have chosen not to do so (I don’t actually recommend doing this).

We have done preliminary analysis of applying our method to gene regulatory networks from FANTOM and GTEX, and found preliminary data that indicate that our method does not work well on these networks. We think this is a combination of regulatory networks being more cell-type specific and the data to generate these networks inherently more noisy. We thank the reviewer for acknowledging that a thorough analysis of applying our method to these other networks is beyond the scope of this paper and have not included this data in the manuscript. Of note, the STRING network, contains multiple interaction types, so we built networks using the different interaction types, and used the cancer GWAS hits to detect candidate core genes. We have described these results in the results section (**lines 542-566**) and the discussion(**lines 650-652**). The results are shown in **Table 2** in the manuscript (shown below). Overall, we found that the physically binding networks described in this manuscript performed the best, but this might be because it has the largest number of nodes.

Type	# nodes	# candidate core genes	Is TP53 a candidate core gene?	Clinvar	Oncokb
Reaction	5583	116	no		
Binding	11,049	109	yes	enriched	enriched
Catalysis	5,277	21	no		
Activation	5,229	35	yes	enriched	
Inhibition	2,802	3	no		
Post-translational Modification	2,566	8	no		enriched
Expression	1,460	55	no		enriched
Co-expression	8,231	167	yes		

3. For any genes that you especially highlight in the text (BRCA1, APP, etc), make sure to be clear about whether they are annotated as GWAS genes in your dataset and also about whether they have been implicated by genetic studies that you did not include. I think a main table could be helpful here: for example, the BRCA1 row could contain a p-value using your method, the rank of this p-value out of all genes, a “no” indicating that this gene is not annotated as a cancer gene in your GWAS data set, and a reference for a study about rare variation in BRCA1.

We thank the reviewer for this suggestion and have included a main table (**Table1**) that clearly captures this information.

Candidate Core Gene	Study accession	P-value	Rank of p-value	GWAS hit	Study with rare variant
BRCA1	GCST001937	0.026	2/21	no	(Hall et al. 1990; Friedman et al. 1994; Miki et al. 1994; Kuchenbaecker et al. 2017)
INS	GCST001213	0.017	1/1	no	(Nishi and Nanjo 2011)
APP	GCST002245	0.039	27/28	no	(Chartier-Harlin et al. 1991; Goate et al. 1991; Murrell et al. 1991)
PCSK9	GCST000282	0.001	6/77	no	(Dron and Hegele 2016; Burke et al. 2017)
SNCAIP	GCST002544	0.011	1/1	no	(Engelender et al. 1999; Engelender et al. 2000)
LPL	GCST000132	0.001	7/83		(Evans et al. 2011)

4. The writing style is idiosyncratic, especially in the methods section. I found it perfectly readable, but it could be good to run this by someone who is a strong writer.

We have made efforts to improve the writing style, especially in the methods section.

Reviewer 2

In “Protein-protein interactions uncover ‘core genes’ within Omnigenic disease networks”, Ratnakumar and colleagues investigate pairing protein-protein interaction with genome-wide

association study (GWAS) data, as curated by the NHGRI/EBI GWAS Catalog, to nominate 'core genes' as conceived by the omnigenic model. The authors hypothesize that core genes will physically interact with GWAS hit genes and develop a graph-based algorithm to call genes that interact with GWAS hits more than expected by chance. They apply this both within individual GWAS and across GWAS annotated with the same GWAS catalog-provided 'parent term'. Significantly interacting genes (which may contain GWAS hit genes) exhibit stronger signals for loss of function intolerance (pLI scores) and stronger enrichment for curated relevant genes and genes implicated via reportedly pathogenic variants in ClinVAR. This work takes a welcome approach to refining the omnigenic model; however, the current manuscript lacks sufficiently precise methods and language to be assured of its conclusions.

We thank the reviewer for their thoughtful summary of our work. We apologize for lack of clarity in some of the language used to describe the results and in the revised manuscript have focused on more precisely defining terms and providing additional details for methods. Responses to specific comments follow below.

1.The distinction between the following terms can get lost: GWAS hits, top GWAS hits, mapped genes, new genes, core genes, interact, etc. Some of this could be clarified in Figure 1 and Figure 2 schematics. It might be easier to follow if you called your candidates as candidate core genes, or hub genes that you later argue serve as core genes. The language interferes with the message. I would argue the phenomena described are more precise than the idea of 'core genes', 'interactions' and other terms. This concreteness should be embraced rather than obscured.

We thank the reviewer for this important perspective. As they have suggested, we have replaced 'core genes' with 'candidate core genes'. We have also renamed the genes into 4 different categories ('PPI only', 'PPI-GWAS', 'GWAS only' and 'All'), and in the somatic analysis we renamed the genes into ('Somatically Mutated candidate core genes', 'PPI-Somatically Mutated' and 'Somatically Mutated'). We have included a definition box (**lines 209-219**, and shown below), which includes definitions for 'GWAS', 'GWAS hit', 'PPI', 'Core Gene', 'Candidate core gene', 'GWAS only', 'PPI-GWAS', 'PPI only', 'All' and 'Somatically mutated candidate core genes', 'PPI-Somatically Mutated' and 'Somatically Mutated'. We have also replaced interactions with 'pp interactions' or 'PPI', and replaced mapped genes with 'PPI-GWAS', replaced new genes with 'PPI only' and have updated the schematic in **Figure 2a** (shown below in **a.** with figure caption) to point out these terms visually.

BOX 1: DEFINITIONS

GWAS: *Genome-wide Association Study*

GWAS Hit: *Gene within genome-wide significant loci (P<5X10^{-8})*

PPI: *Protein-Protein Interaction*

Core Gene: *Gene with direct roles in disease*

Candidate Core Genes: *Genes identified by our method that we think are likely to be core genes*

GWAS only: *GWAS hits that are not candidate core genes*

PPI-GWAS: *GWAS hits that are also candidate core genes*

PPI only: *Candidate core genes that are not GWAS hits*

ALL: *All 11,049 genes within the STRING network*

Somatically Mutated Candidate Core Genes: *Candidate core genes predicted from somatically mutated cancer genes*

PPI-Somatically Mutated: *Somatically mutated cancer genes that are also candidate core genes*

Somatically Mutated: *Somatically mutated cancer genes that are not candidate core genes*

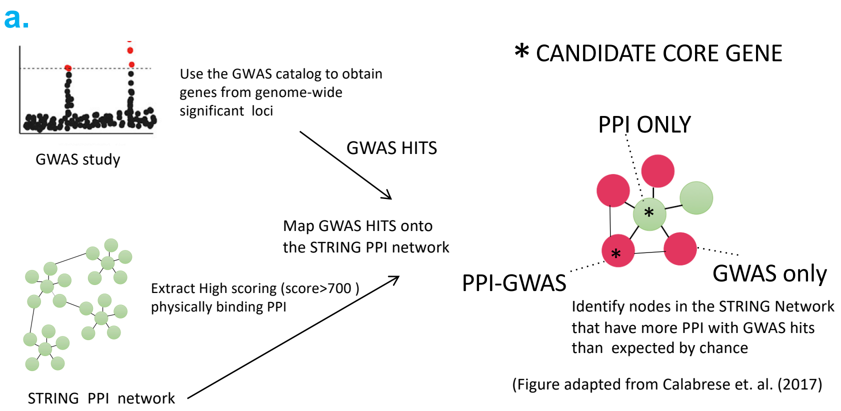

Fig. 2:

a. This figure was adapted from(Calabrese et al. 2017), it depicts our method for detecting candidate core genes, which involves putting GWAS hits from the GWAS catalog onto the STRING PPI network, and identifying neighboring nodes that have excess PPI with the GWAS hits. We also define the following terms. 'GWAS': Genome-wide Association Study, 'GWAS HIT' Gene within genome-wide significant loci (P<5x10^{-8}), 'PPI': Protein-Protein Interaction. 'CORE GENE': Genes with direct roles in disease biology 'CANDIDATE CORE GENE': Genes identified by our method that are likely to be core genes. 'GWAS ONLY': GWAS hits that are not candidate core genes. 'PPI-GWAS': GWAS hits that are also candidate core genes. 'PPI only': candidate core genes that are not GWAS hits. 'All': all 11,049 nodes within the STRING network.

2.As far as I can tell, many statistical tests are not controlled for node degree. See **Figure 3e**, lines 431-436, 443, 475-477, 721-727.

<u>Figure 3e</u>

Below is the original **Figure 3e**

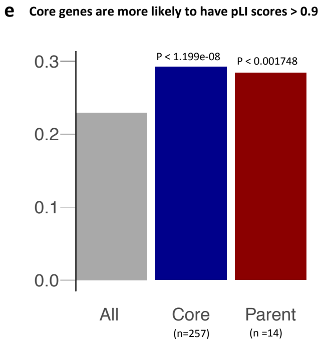

We thank the reviewer for bringing this to our attention. We have repeated the pLI analysis, but this time we binned the nodes by degree ranging from (1-50, 50-100, 101-150, 150-200, >201), and after binning we did not find any strong enrichment in pLi in candidate core genes (see below), and as a result have removed this analysis from the manuscript.

pLI Individual GWAS studies

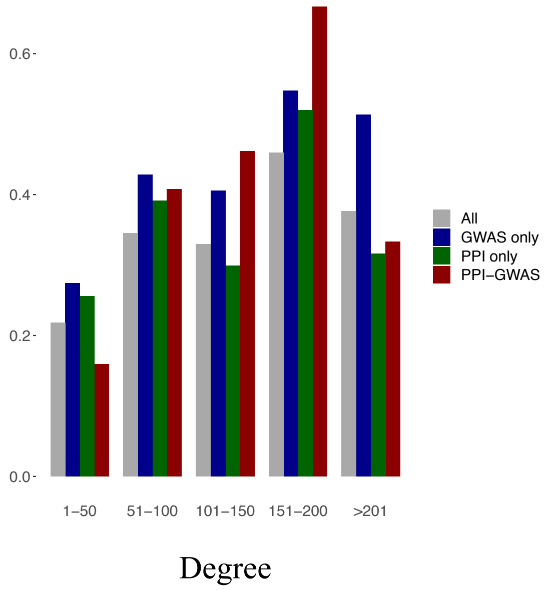

pLI Parent Terms

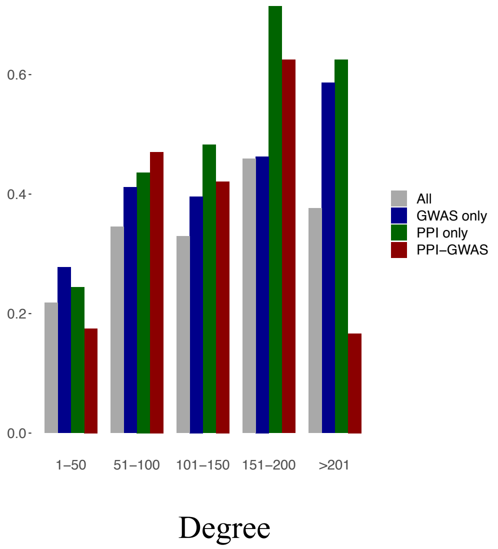

Lines 431-436

Shown below are **lines 431-436** from our original submission:

We tested for evidence of increased PPI among these somatically mutated, genes by comparing the PPI on the STRING PPI network to 50,000 randomized networks and observed 495 PPI

Here, we describe how we estimated whether the 218 somatically mutated cancer genes have more PPI with each other than expected by chance. One concern might be that the 218 somatically mutated genes have higher degree distribution than the rest of the network. However, this shouldn't influence our analysis, because we are not comparing the 218 somatically mutated genes to the rest of the network, instead, we are comparing the 218 somatically mutated genes on the STRING network to the same 218 somatically mutated genes on each of the 50,000 randomized networks (see below). The randomized networks have the same topology and degree distribution as the STRING network and were built following the procedure set out by (Rossin et al. 2011).

218 somatic genes -> on STRING network -> # of PPI between somatic genes = 495
218 somatic genes -> Randomized network1 -> # of PPI between somatic genes = 278
218 somatic genes -> Randomized network2. -> # of PPI between somatic genes = 300
Randomized network3
Randomized network4
Randomized network5
Randomized network6
Randomized network7
Randomized network8
.
.
.
Randomized Network 50,000

After putting the 218 somatically mutated genes on each network, we counted the number PPI between the 218 somatically mutated genes, then compared the number of PPI between the 218 somatically mutated genes on the real STRING network to the distribution of the number of PPI between 218 somatically genes across 50,000 randomized networks. The number of PPI edges we observed on the real STRING network was 495, while the largest number of PPI edges we observed on any of the 50,000 randomized networks was 366. Suggesting that the 218 somatically mutated cancer genes have more PPI between them than expected by chance. We have endeavored to make this clearer in our methods section (**lines 1004-1026**).

Line 443

Shown below is line 443 from our original submission:

We found that out of 11,049 nodes in the PPI network, TP53 was the second most enriched in terms of the number of direct PPI with the somatically mutated cancer genes

We used the 218 somatically mutated cancer genes to detect candidate core genes. We wanted to find proteins that are "hubs" or "master-regulators" of these 218 somatically mutated cancer genes. We applied our same candidate core gene detection method, but instead of GWAS hits we used the 218 somatically mutated cancer genes. One of the candidate core genes we predicted was *TP53*, when we compared the 218 somatically mutated cancer genes to *TP53*, we obtained the following numbers:

(1) Degree of node *TP53*: 284
(2) Number of somatically mutated cancer genes: 218
(3) Overlap between (1) & (2): 31

This means that *TP53* has direct PPI with 284 nodes and 31 of these are somatically mutated cancer genes. Given the degree of 284 for *TP53,* an overlap of 31 out of a total of 218 somatically mutated genes is much more than you would expect by chance, that's why we get a highly significant p-value (p-value = 1.31E-11). However, *TP53* was only the second most significant in terms of p-value, *CTNNB1* had the most significant p-value (p-value = 5.11E-12).

<u>**Lines 475-477**</u>

Shown below are **lines 475-477** from our original submission:
Consistent with this, is the finding that top GWAS hits that detect core genes have more PPI with each other than expected by chance, and that core genes tend to have higher degree than other nodes in the network

<u>**Line 721-727**</u>

Shown below are **lines 721-727** from our original submission:
So we compared all of the nodes from the 270 studies detected by 2 or more top GWAS hits and counted how often these top GWAS hits interact to how often the top GWAS hits that detect each core genes interact. We found that top GWAS hits that detect core genes are more likely to interact with each other.

We thank you for pointing out that this comparison requires us to take node degree into account. When we compared node degree, we found that GWAS hits that detect candidate core genes tended to have higher degree than GWAS hits that do not. This suggests, that our finding that GWAS hits that detect core genes have more PPI with each other than expected by chance is likely due to them having higher degree. Thank you for bringing this to our attention, we have removed this analysis from the paper.

If I understand the methods, the studies are ascertained to have multiple hits, but I believe this creates a bias in the results in favor of studies that are more likely to have more hit nodes and edges, whereas the all node set will remain at the level of typical connectedness. I recommend subsampling nodes of matched degree AND such that there is at least one edge between them, or removing the requirement altogether. Then determine the significance empirically by creating a null distribution from the samplings.

We apologize for not being clearer regarding the methods on study ascertainment, but are quite confident that we controlled for network structure. Importantly, we DID NOT ascertain studies by examining how many GWAS hits they had. We did place a simple filter to remove studies with less than 2 GWAS hits because identifying excess PPI in this case would not be feasible. Below we have compared the number of GWAS hits and degree of GWAS hits at each step of our methods to demonstrate that significance is not driven by differences in GWAS hits compared to all other genes.

To describe our study selection process in more detail, we first started with 1,381 GWAS studies, and after removing studies with only 1 GWAS hit, we were left with 968 studies. Out of these 968 studies only 365 studies had at least 1 PPI edge between their GWAS hits. To determine what proportion of the studies with at least 1 PPI edge had more PPI edges than expected by chance, we compared the number of PPI edges between GWAS hits on the real STRING network to the number of PPI edges between GWAS hits on 50,000 randomized networks. We deemed GWAS studies whose GWAS hits had more PPI edges on the real STRING network than observed in >95% of the 50,000 randomized networks, as studies with excess PPI between their GWAS hits. In our analysis, we found that out of 365 GWAS studies with at least 1 PPI edge, 270 GWAS studies had more PPI edges between their GWAS hits on the STRING network.

Our method, has 2 stages of filtering. The first filters for studies with at least 1 PPI edge (Stage 1). The second filters studies with at least 1 PPI edge, for studies with excess PPI edges (Stage 2). We have compared the number of GWAS hits and the degree of GWAS hits at each of these two filtering stages.

Stage 1: compare studies with at least 1 PPI edge (n=365) vs. studies with no PPI edges (n=968-365)
Stage 2: compare studies with excess PPI edges (n=270) vs. studies without excess in PPI edges (n = 365-270)

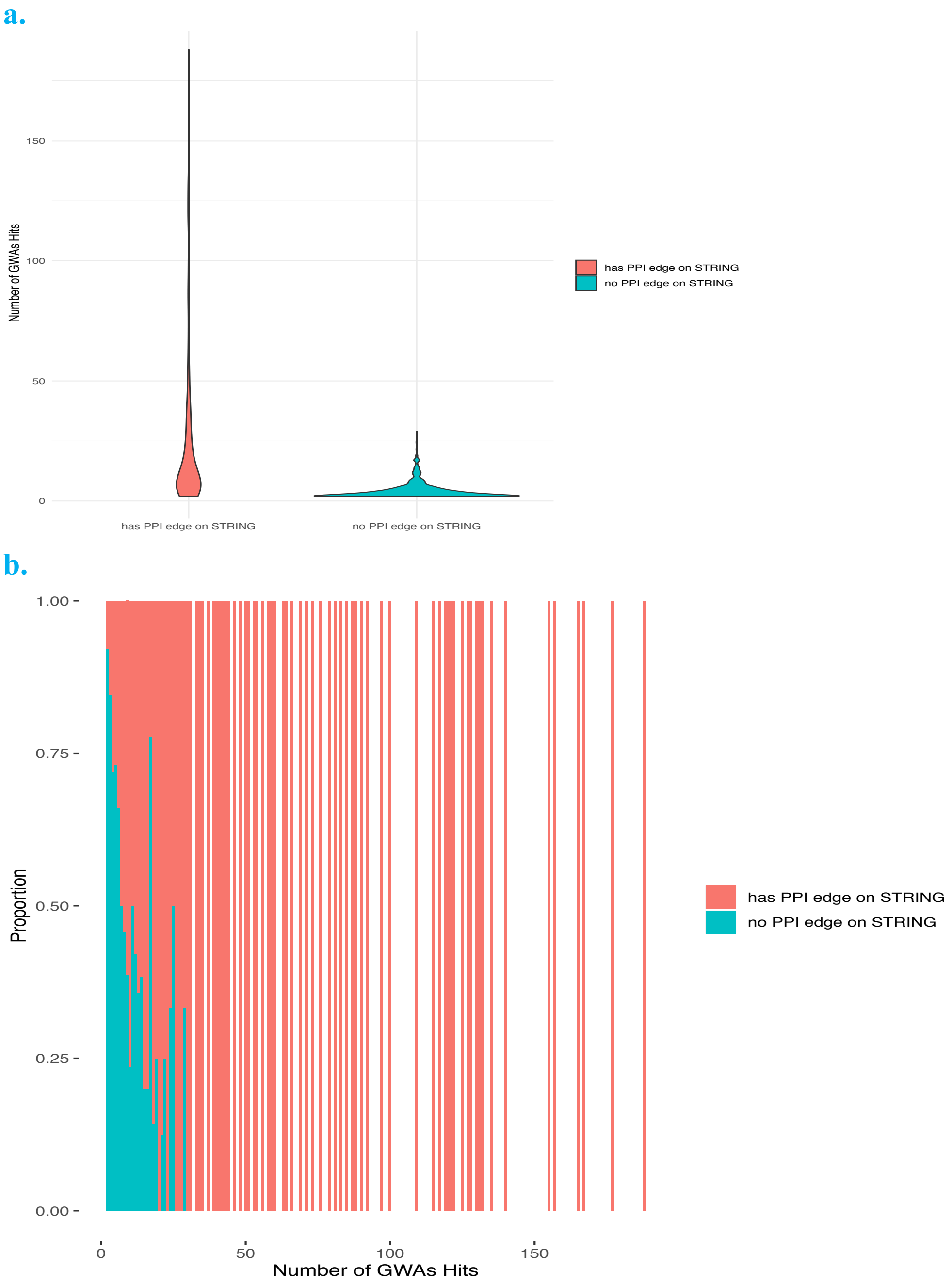
a.
Number of GWAs Hits
150
100
50
0
has PPI edge on STRING
no PPI edge on STRING
has PPI edge on STRING
no PPI edge on STRING
b.
1.00
0.75
0.50
0.25
0.00
Proportion
0
50
100
150
Number of GWAs Hits
has PPI edge on STRING
no PPI edge on STRING

Stage 1: compare studies with at least 1 PPI edge (n=365) vs. studies with no PPI edges (n=968-365)

We compared degree distributions and found that the mean degree of GWAS hits in studies with at least 1 PPI edge was 28.839, while the mean degree of GWAS hits in the studies with no PPI edges was 24.234, and the difference was statistically significant (p<2.583e-08, t.test). We then compared the number of GWAS hits and found that the mean number of GWAS hits in the studies with at least 1 PPI edge was 26.838, while the mean number of GWAS hits in the studies with no PPI edges was 4.33(see **a.** above).

We then plotted the proportion of studies that have at least 1 PPI edge at each number of GWAS hits(see **b.** above), and demonstrate that at low numbers of GWAS hits, the likelihood of having at least 1 PPI edge between the GWAS hits is low, but as the number of GWAS hits increases the chances of having at least 1 PPI edge between the GWAS hits increases.

Although we did not specifically filter studies based on the number of GWAS hits, when we filter by whether the study contains a PPI edge or not we inadvertently enrich for studies with more GWAS hits. **However, this does not affect our analysis because this is just the initial filtering step and it is only at the next filtering step that we identify studies with excess PPI edges**. We will show below that no bias exists between the degree of GWAS hits and number of GWAS hits at the next stage.

c.

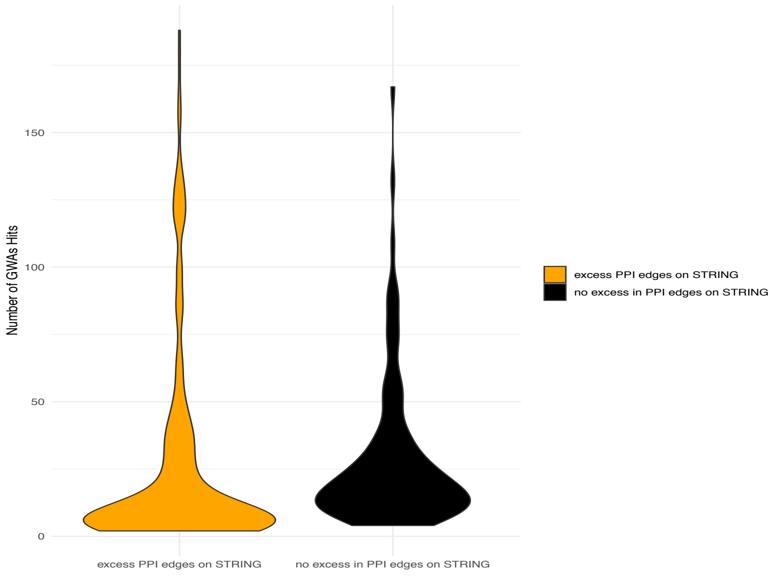

d.

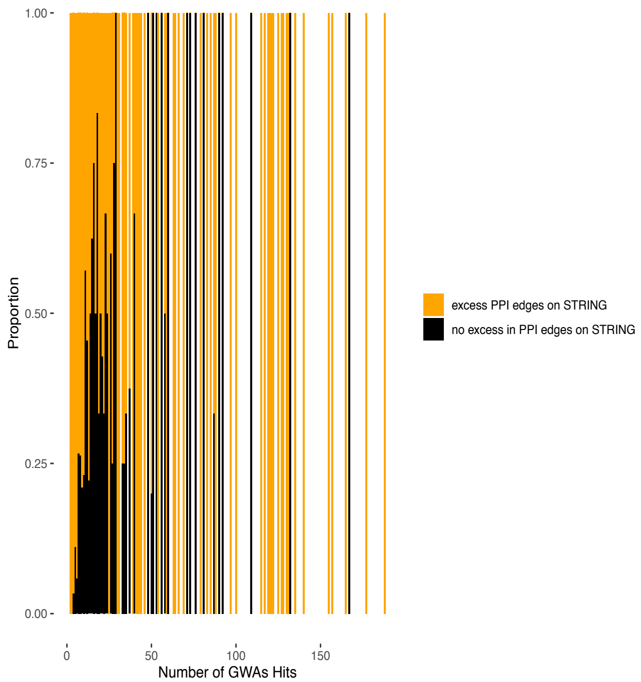

Stage 2: compare studies with excess PPI edges (n=270) vs. studies with no excess in PPI edges (n = 365-270)

We compared the degree of GWAS hits and the number of GWAS hits between the 270 studies in which we detect excess PPI edges to the 95 studies with no excess in PPI edges. We found no statistically significant difference between the mean degree of GWAS hits (28.405 vs. 29.969, p-value = 0.1169, t.test), and found no statistically significant difference between the number of GWAS hits(26.196 vs. 28.663, p-value = 0.5096, t.test) (see **c.** above).

We plotted the proportion of studies with excess PPI edges at each number of GWAS hits(see **d.** above), and found that although there is a tendency for studies with low number of GWAS hits to have no excess in PPI edges, there are many studies with large numbers of GWAS hits that do not have excess PPI edges. The table below shows studies with 50 or more GWAS hits without excess in PPI edges between them.

Study_Accession	Disease_Trait	Number_of_GWAS_hits
GCST001337	Platelet_count	53
GCST001956	Height	58
GCST002541	Menarche_age_at_onset	92
GCST002702	Height	73
GCST003676	Educational_attainment_years_of_education	60
GCST004601	Red_blood_cell_count	132
GCST004604	Hematocrit	81
GCST004607	Plateletcrit	167

GCST004615	Hemoglobin_concentration	87
GCST004622	Reticulocyte_count	109
GCST005232	Neuroticism	56
GCST005951	Body_mass_index	90
GCST005984	Glomerular_filtration_rate	51
GCST005985	Creatinine_levels	50
GCST005991	Platelet_count	71
GCST006011	Mean_corpuscular_volume	76

Since we find no difference in the number of and degree of GWAS hits from studies with excess PPI edges compared to studies without, suggests that whether a study has excess PPI edges or not, is not driven by differences in the number of and degree of GWAS hits. We have described these results in **lines 150-156**.

If the significance is driven primarily by differences in degree between GWAS hits and all other genes, that might be a result in its own right, but the interpretation would change.

e.

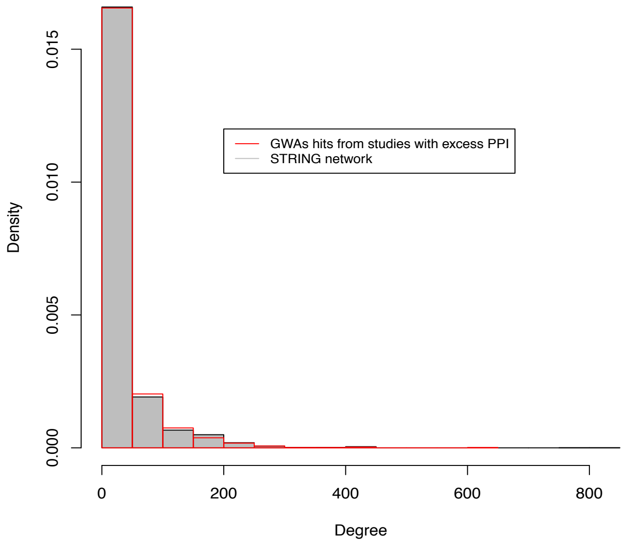

We also compared the degree distribution of GWAS hits from studies with excess PPI to the degree distribution of the whole network (see **e.** above), and found little difference. The mean degree of GWAS hits from studies with excess PPI was 29.225 while the mean degree of the whole STRING network was 29.538, and the difference was not statistically significant (p-value = 0.7736, t.test). So, the significance of excess PPI edges among GWAS hits is not due to the differences in degree of GWAS hits compared to the rest of the network.

3. The procedure in 660-681 is hard to follow. I do not understand the two separate steps of Benjamini-Hochberg correction and empirical FDR calculation. It also sounds like the empirical FDR is a p-value, not an FDR. Is it not enough to create one null distribution and

then correct for multiple tests on the resulting p-values? This more complicated route, if preferable, requires justification.

The reviewer is correct, we are providing empirical p-values not an FDR. To try and be as conservative as possible, we performed Benjamini-Hochberg correction followed by empirical p-value – which may have made the description of the method less clear. However the empirical p-value step didn't significantly alter the results in terms of identifying candidate core genes, so we have taken the advice of the reviewer and have redone the analysis to remove the empirical p-value step. We now deem nodes with Benjamini-Hochberg adjusted p-value <0.05, to be candidate core genes. We have also rewritten our methods (**lines 801-814**) to make it easier to follow.

4. In lines 333-340 and the legend for Figure 3, you state that the coincidence of core genes and GWAS hits supports the lesser polygenicity of lipid traits. This is repeated in the discussion (line 481). Why this should be the case is unclear to me. There may be a case for polygenicity here but it needs to be fully reasoned to merit inclusion.

Thank you for your comments, we included this in the manuscript because we thought it was interesting that other groups found Lipid traits to be less polygenic, and we found that Lipid traits have the highest proportion of PPI-GWAS amongst candidate core genes. However, other groups used genome-wide heritability estimates to come to their conclusion of reduced polygenicity, and we have not formally assessed for this, so have removed our comments of the lesser polygenicity of lipid traits, from the figure legend, results section and discussion section. Our finding of more PPI-GWAS amongst lipid trait candidate core genes, demonstrates that GWAS are better able to detect candidate core genes in lipid traits, which could be due to better phenotyping or larger sample sizes. (**Lines 394-398** in the manuscript).

5. I am currently not persuaded by the content of most of Figure 4, partially because of the points above. With respect to 4a, I do not understand the significance of immune genes forming a cluster or how that determination was made (the nodes are not marked to distinguish the immune genes). With respect to 4e, it is not enough to say TP53 has more signal than other high degree genes – is this statistically significant? There is an apparent trend (hard to see the way it is plotted) for high degree nodes to have more overlap, so this has to be evaluated more carefully. This section of the manuscript does not concisely communicate the insights made possible by this approach and goes largely unexamined in the Discussion.

With respect to 4a, I do not understand the significance of immune genes forming a cluster or how that determination was made (the nodes are not marked to distinguish the immune genes).

Below is **Figure 4a** from our original submission

a.

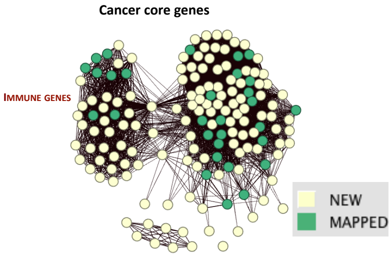

Below is the current version of the figure (it is now **S4 Figure b**)

b.

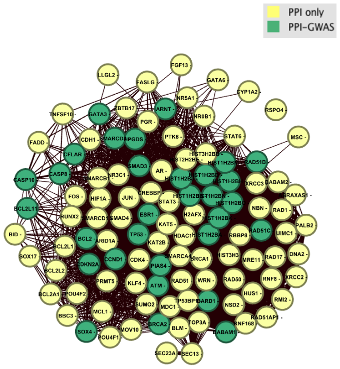

We apologize that the method of clustering was not clear in **Figure 4a**. The clustering was performed by evaluating the PP interaction partners shared between each pairwise combination of cancer parent term candidate core genes. The number of PP interaction partners shared between every node pair, was stored as a weight and the force directed layout algorithm known as Fruchterman Reingold (Fruchterman and Reingold 1991) within the Gephi program was used to cluster the nodes using these weights (see above **a.**).

Due to comments from this reviewer and another, we more carefully examined the GWAS hits that were identifying the candidate core genes within the immune cluster, and this led us to develop additional filters on our analysis. Namely GWAS hits that detect candidate core genes cannot be within 1Mb of each other. We implemented this filtering to remove cases where candidate core genes are detected by GWAS hits from the same locus. After applying this filter many of the HLA candidate core genes were filtered out, and as a result we no longer have an immune cluster amongst the cancer parent term candidate core genes (see above **b.**).

With respect to 4e, it is not enough to say TP53 has more signal than other high degree genes – is this statistically significant?

Shown below is **Figure 4e** from our original submission:

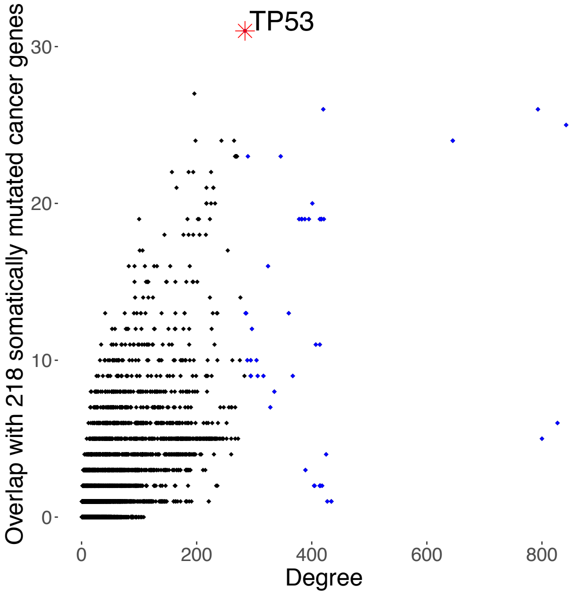

Yes, *TP53* is statistically significant (p = 1.31E-11). *TP53* has degree of 284 and overlaps with 31 out of the 218 somatically mutated cancer genes.

There is an apparent trend (hard to see the way it is plotted) for high degree nodes to have more overlap, so this has to be evaluated more carefully.

Thank you for your comment. You are correct that higher degree nodes do have more PPI with the 218 somatically mutated cancer genes. We have made this plot again(**Figure 5d,** and shown below), but this time we took every node in the network (n=11,049) and counted the number of PPI edges each node has with the 218 somatically mutated cancer genes. In order to better understand how the degree of the node influences the number of PPI edges, we plotted the degree of the node vs. the number of PPI edges with the 218 somatically mutated cancer genes (see below **a.**), and have shown the candidate core genes in red. It is clear, there is positive correlation between the degree of the node and the number of PPI with the 218 genes, however, all the candidate core genes are in the top half of the plot, demonstrating that the candidate core genes have more PPI with the 218 genes, than we might expect given the degree.

a.

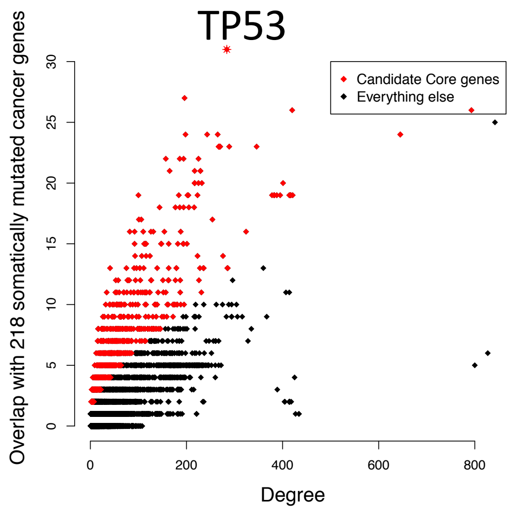

b.

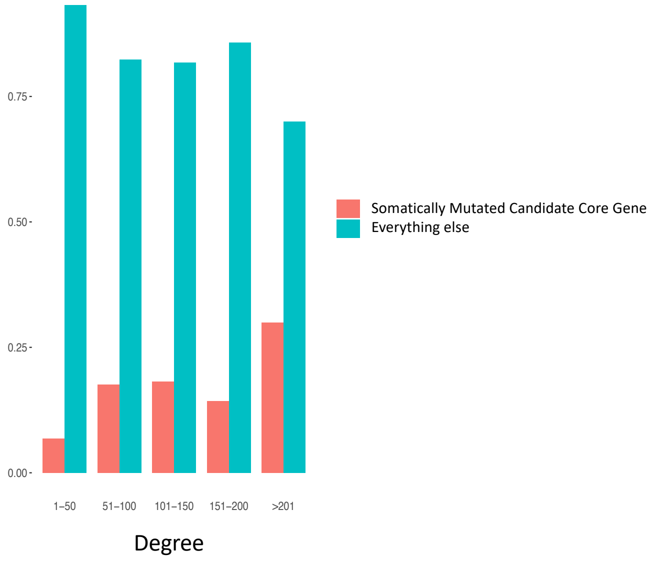

We also binned all the nodes in the network (n=11,049) (see **b.** above) into bins with degree range of (1-50, 51-100, 101-150,151-200, >201), and have shown the proportion of nodes within each degree bin that are candidate core genes, and found that the proportion of candidate core genes is only slightly higher amongst nodes with high degree (>201) (**S6 Figure**).

This section of the manuscript does not concisely communicate the insights made possible by this approach and goes largely unexamined in the Discussion.

There are a number of unique insights made possible by this approach, these include:

(1) Our finding of 109 cancer parent term candidate core genes, and 843 somatically mutated candidate core genes
(2) Our demonstration that somatically mutated cancer genes are connected to each other with excess PPI, suggesting an underlying biological process such as a protein complex unifying the seemingly disparate somatically mutated cancer genes
(3) Our demonstration that *TP53* is a highly statistically significant PPI-Somatically Mutated candidate core gene, suggesting that in addition to being somatically mutated itself, it may also be the mechanism by which other somatically mutated cancer genes confer risk

We have updated the results section (**lines 519-526**) and the discussion (**lines 589-598**) with the unique insights made possible by our method.

6. More broadly, the manuscript is lacking clear proof of usefulness of the work beyond nominating promising genes. I am not convinced this work will be truly impactful without such a demonstration. One possibility is to examine the 'coreness' of cancer drug targets or compare this analysis to other published measurements of interest such as heritability (various sources) or polygenicity (O'Connor 2019, AJHG).

In order to assess the 'coreness' of cancer drug targets, we obtained cancer drug targets from Oncokb(Chakravarty et al. 2017) (there were 52 cancer drug targets that were also within our list of 11,049 nodes), and then looked for overlaps with cancer candidate core genes in two separate analyses (germline and somatic).

a.

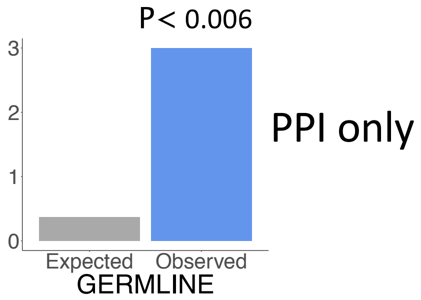

In the germline analysis we compared the 52 Oncokb(Chakravarty et al. 2017) cancer drug targets to the cancer parent term candidate core genes, and found enrichment in PPI-GWAS compared to GWAS only (13% vs, 2%, P< 0.006), and PPI only (4% vs 0.5%, p< 0.006) and PPI-GWAS (13% vs 0.5%, p< 1.469e-05) compared to the whole network (**Figure 4e** in the manuscript and shown above in **a.**) We have described these results in the results section(**lines 429-434**), and the discussion section (**lines 585-588**).

b.

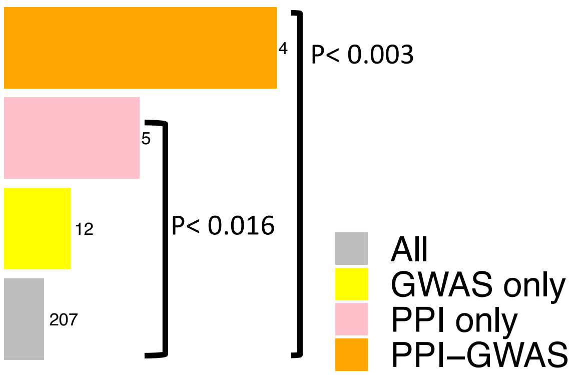

We also obtained drug targets from the Therapeutic Targets Database(Wang et al. 2020), and extracted cancer drug targets by filtering for disease entries containing the words 'cancer', 'tumor' or 'Metastatic', and found 207 cancer drug targets that were also within our list of 11,049 nodes. Amongst these 207, we found PPI-GWAS was enriched compared to GWAS only (13% vs. 3.1%, p< 0.025), and both PPI only (6.4% vs. 1.8%, p< 0.016) and PPI-GWAS (13% vs. 1.8%, p< 0.003) were enriched compared to the rest of the network. We have described these results in the results section (**lines 434-439**) and the discussion section (**lines 585-588**).

c.

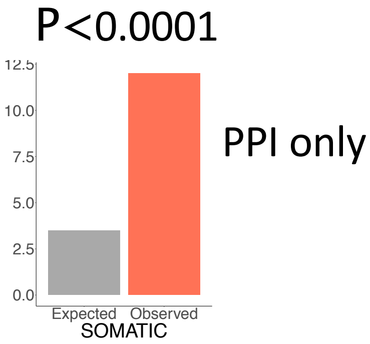

In the somatic analysis we compared the 52 Oncokb(Chakravarty et al. 2017) cancer drug targets to the 843 somatically mutated candidate core genes. We found that the PPI-Somatically Mutated candidate core genes were enriched compared to Somatically Mutated (24% vs. 7% p<0.001) and found that PPI only (1.6% vs. and 0.5%, p<0.001) was enriched compared to the whole network(**Figure 5f** in the manuscript and shown above in **c.**). We have described these results in the result section **(lines 532-535**) and in the discussion section (**lines 585-588**).

d.

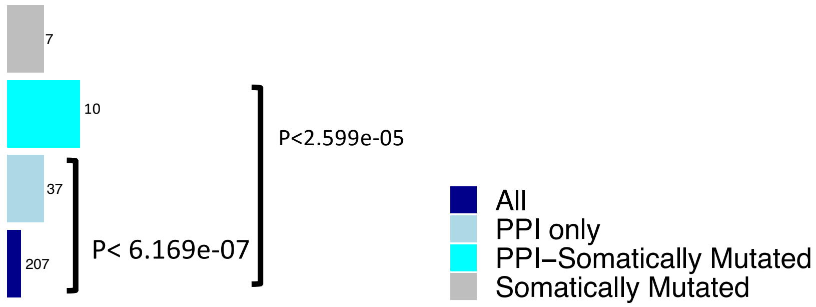

We performed a similar analysis using cancer drug targets from the Therapeutic Target Database(n=207) (Wang et al. 2020) and found similar results (**Figure 5g**). We found both PPI only (5% vs. 1.8%, p< 6.169e-07) and PPI-Somatically Mutated (9.9% vs. 1.8%, p< 2.599e-05) was enriched compared to the whole network (**Figure 5g,** see **d.** above**).** We described these results in the results section (lines 535-540) and discussion section (**lines 585-588**).

Heritability

We thank the reviewer for this interesting suggestion, and agree that enriched heritability could provide evidence for the 'importance' of candidate core genes. However, we were unsure how to do this. We downloaded per SNP heritability estimates from the UK biobank (https://nealelab.github.io/UKBB_ldsc/) for phenotypes similar to the GWAS phenotypes, and then extracted per SNP heritability estimates from common variants (MAF>0.05) that overlap the genes in GWAS only, PPI only, PPI-GWAS, all 11,049 genes in our network and all common SNPs. Then for each list of genes we summed across the absolute value of the per SNP heritability values, then divided by the total number of SNPs in that category (see table below). In the 'Lipid and Lipoprotein Measurement' parent term, we found that the SNPs in PPI-GWAS had higher per-SNP heritability than the SNPS in GWAS only. However, we found little evidence that PPI only genes are enriched for heritability compared to other genes in the genome. Below we show results for selected parent terms.

Parent term	UK biobank phenotype	GWAS only	PPI only	PPI-GWAS	11409 genes	All SNPs
Lipid_or_lipoprotein_measurement	Cholesterol	2.860	0.912	5.012	1.029	0.993
Cancer	Breast Cancer	0.973	0.890	1.312	0.833	0.828
Cancer	Prostate Cancer	0.989	0.822	0.900	0.837	0.839

Hematological_measurement	Mean platelet volume	2.458	1.462	2.438	1.192	1.203
Digestive_system_disorder	Diseases of the digestive system	0.955	0.955	0.999	0.891	0.886
Cardiovascular_disease	Major coronary heart disease event	1.065	0.903	0.703	0.859	0.849

Polygenicity

We thank the reviewer for this suggestion, however because the main goal of this manuscript is to find core genes, we think that comparing candidate core genes to polygenicity measures is beyond the scope of this paper.

2.12. Reviewer comment

7. If the authors are arguing that physical interactions are uniquely positioned to reveal core genes from GWAS hits, it seems that the method should be applied to coexpression or other interaction types for the sake of comparison.

In this manuscript we only considered physically binding protein-protein interactions (by requiring that the 'mode' column of the 9606.protein.actions.v10.5.txt file was set to 'binding'). In the FAQ section of the STRING network website (http://version10.STRING-db.org/help/faq/), it states that these refer to physical interactions. However there are 6 other types of entries in this field, which include (reaction, catalysis, activation, inhibition, ptmod(post translational modification) and expression). We also noticed that the 10th column of the file 9606.protein.links.full.v11.0.txt was named 'coexpression', when we extracted these interactions and applied a score cut-off of 700, we were left with only 590 nodes, and with no score filtering were left with 8,231 nodes.

We built networks from these 7 other interaction types and used the 403 cancer parent term GWAS hits to detect cancer parent term candidate core genes across the different networks. For comparison purposes we filtered all of the networks for the 11,049 nodes present in the "binding" network we used in the manuscript. To assess performance we checked whether *TP53* was detected as a candidate core gene, whether the candidate core genes were enriched for Clinvar pathogenic variants and/or enriched for cancer drug targets from Oncokb. The results are summarized in **Table 2** below. Comparison across networks revealed that the binding network we used in or manuscript outperformed other networks. However, this might be, because this interaction type had the largest number of nodes.

Type	# nodes	# candidate core genes	Is *TP53* a candidate core gene?	Clinvar	Oncokb
Reaction	5583	116	no		
Binding	11,049	109	yes	enriched	enriched

Catalysis	5,277	21	no		
Activation	5,229	35	yes	enriched	
Inhibition	2,802	3	no		
Post-translational Modification	2,566	8	no		enriched
Expression	1,460	55	no		enriched
Co-expression	8,231	167	yes		

Minor Points

1. I have trouble understanding some parts of the manuscript:

- **Line 171**
- **Line 215-216**
- **Line 221-223**
- **Line 253**
- **Line 588 (By SNP platform do you mean genotyping array?)**

Yes, by SNP platform we mean genotyping array. This information is found in the description of the headers of the GWAS catalogs is in this URL https://www.ebi.ac.uk/gwas/docs/fileheaders.

"PLATFORM (SNPS PASSING QC)*: Genotyping platform manufacturer used in Stage 1; also includes notation of pooled DNA study design or imputation of SNPs, where applicable"

- **Line 601-606**

We have rewritten the lines above to improve the writing style.

2. Cut down and clarify some repetitive or long sections, including:

- Line 312-316
- Line 319-320
- Line 401-402
- Line 406-411
- Figure legends in general. Avoid getting into the results in the legend text.

We have rewritten the lines above to improve the writing style.

3. There are a number of sentence fragments that can be fixed, including:

- **Line 244**
- **Line 338**
- **Line 436**
- **Line 621**

We have fixed the sentence fragments in the lines above.

4. Text is often too small to read:

- **Figure 2 b, d**
- **Figure 3 b-e**
- **Figure 4 e**

We have increased the font size in the figures above.

5. Throughout the text, genome-wide association studies are abbreviated as GWAS, but GWAS (with a capital S) is more standard in the field. I advise making this substitution.

We have replaced every instance of GWAS with GWAS throughout the text.

6. Other line items:
229-237: The parenthetical remarks are fairly long and do not mix well with references.
We have removed the parenthetical remarks.
248: Remove the double tab
We have removed the double tab.
358: you mean 3e, not 3c, I believe.
We have fixed this.
365/422: Use of 'new' to describe interacting genes is confusing. Use 'tagged' and 'untagged' or 'tagged' and 'interactor' or something that better captures the class of genes you are describing
We changed "new" to PPI only and changed mapped to PPI-GWAS.
406: Fix formatting of "et al."
We have fixed this.
572: How are physically interacting genes identified? I am not sure that this is specified in STRING, and if it is, please state which file/column you consulted. I know there is an experimentally determined column; that column encompasses many types of assays, so I advise explaining the scope.

The way we obtained physically interacting genes was by requiring that the "mode" column of the proteins.actions.txt file was set to "binding". This is outlined in the FAQ section of the STRING network website. We have included a screenshot of this below.

> **I want to differentiate physical interactions from functional ones within STRING**
>
> In order to get the physical interactions you need to download proteins.actions.(version).txt.gz from download section. If the interaction is marked as "binding" you can be sure that this is a physical interactions. If interaction does not have "binding" specified (i.e., antyhing else) it may be either physical or functional.

593: I believe the p_value_text column is the p value reported in the text. Thus the filtering step described does not immediately make sense to me. It would be best to elaborate.

The description of the headers of the GWAS catalogs is in this URL https://www.ebi.ac.uk/gwas/docs/fileheaders

The header description for P-value (text) is as below:
"P-VALUE (TEXT)*: Information describing context of p-value (e.g. females, smokers)."

P_value_text refers to the context of the p-value. Most entries in the P-VALUE (TEXT) entry are empty, indicating that the p-value refers to the whole dataset. The instances where it is not empty are instances where p-values are calculated on a subset of the data. The different entries in this column are listed below, with the number referring to the number of entries. An empty STRING is the most common entry in this column.

Reviewer 3

Reviewer #3: The manuscript by Ratnakumar and colleagues presented results analyzing GWAS associated genes in light of protein-protein interaction (PPI) networks. Their hypothesis is that genes physically interacting with disease-associated genes with high frequencies are candidates of "core genes", according to the omni-genetic model of complex trait genetics. Their analysis identified hundreds of putative "core genes" across a large set of phenotypes.

While I think some of the results, especially those about cancer, are interesting, I am not convinced that their analysis really sheds light on core genes under the omnigenetic model. Also the overall novelty of the work is questionable to me. Below are specific comments:

Major comments

1) Core genes have a specific meaning under the omnigenetic model: they are believed to mediate the effects of a potentially large number of genes associated with a complex trait (called "peripheral genes"). The omnigenetic paper discusses regulatory influence of perturbation of peripheral genes on core genes, in partiular, as one main mechanism. It seems to me that this paper mis-interprets the concept of core genes. All the analysis are based on the numbers of PPIs: there is no evidence that the genes interacting with other GWAS genes would "mediate" their effects on disease risks.

We thank you for your thoughtful and constructive feedback. We agree with you that under the omnigenic model peripheral genes that have significant disease associations

are thought to mediate their risk by weak influence on core genes, which are assumed to be central in key disease pathways. Below, we note the definition of core genes directly from the omnigenic paper(Boyle et al. 2017)

"First, we assume that most traits can be directly affected by a modest number of genes or gene pathways with specific roles in disease etiology, as well as their direct regulators (Chakravarti and Turner, 2016). We refer to these as "core genes". Such genes will tend to have biologically interpretable roles in disease, such as the roles of IRX3 and IRX5 in controlling adipocyte differentiation, with consequent effects on obesity (Claussnitzer et al., 2015), or the role of the C4 genes on synaptic pruning in development, thereby affecting schizophrenia risk (Sekar et al., 2016). Furthermore, when core genes are damaged by loss-of-function or other particularly damaging mutations, we can anticipate that these will tend to have the strongest effects on disease risk (although the actual degree of increased risk conferred by the largest effect-size mutations varies greatly across traits (Krumm et al., 2015, Marouli et al., 2017)). "

In the context of this paper we define core genes to be genes with direct roles on the phenotype, and we have included these in the definition box (**lines 209-219**), and in the introduction (**lines 87-88**). It is true that the omnigenic paper discusses regulatory influence of perturbation of peripheral genes on core genes, however, the omnigenic paper also states that the nature of what connects core genes to peripheral genes has not been fully elucidated.

"At this time, our understanding of cellular regulatory networks remains incomplete, but the relevant connections likely include all layers of interactions among cellular molecules, including transcriptional networks, post-translational modifications, protein-protein interactions, and intercellular signaling (Furlong, 2013). "

"If most genetic variants act through cellular networks, then what mediates these connections? Transcriptional regulation, post-translational modification, protein-protein interaction, and intercellular signaling may all contribute. What is the nature and frequency of long-range interactions in cellular networks? How do network architectures vary across cell types and tissues? "

All the analysis are based on the numbers of PPIs: there is no evidence that the genes interacting with other GWAS genes would "mediate" their effects on disease risks.

We thank you for this comment. It is correct that there is no evidence that the genes interacting with other GWAS genes would "mediate" their effects on disease risks. That is why our approach is novel. Currently, the suggested methods for detecting core

genes is deep sequencing and network inference. Our approach of detecting core genes via GWAS hits is novel.

The analysis is more like finding "hub" genes in PPI network. There is a large literature on this topic, including some in the context of disease genetics, e.g. 21764832, 21490723, 25915600. It seems to me that none of these papers is properly cited. Given this background, I think the conceptual novelty of the work is quite limited.

Thank you for bringing these 3 papers to our attention. We agree that they provide important context for our work and have now cited them in the introduction (**lines 99-116**).

Both Chen et. al and Greene et. al use network topology to re-rank GWAS hits, while Wang et. al is a review paper. Chen et. al use a Markov random field to leverage the topology of pathways to re-prioritize GWAS hits, while Greene et. al uses tissue specific networks and support vector machines (SVM) to learn network features of GWAS hits and then compute a probability of disease association for every gene. Wang et.al describes methods for identifying new disease associated genes using distance to known disease associated genes.

The similarities between these papers and our work are that they all integrate GWAS with networks, and use network topology to leverage the idea that functional related proteins tend to reside close together in the network. However, there are a number of differences that make our work novel.

The biggest difference is that we use GWAS hits to find new candidate core genes, and subsequently show that these genes are disease-related and have properties consistent with core genes as defined by the omnigenic model. While the other studies primarily focus on re-ranking GWAS hits. The aims of Chen et. al and Greene et. al were to overcome the challenges of GWAS namely small effect sizes, low statistical strength due to epistasis and missing heritability. However, our aim, was to detect core genes as defined by the Omnigenic model. Another point of difference is that we use high scoring (>700) physically binding protein-protein interactions, while the other papers use pathways and tissue specific networks. Another difference is that the methods in Chen et.al and Greene et. al involve difficult to interpret Math like Markov Random field and SVM. It's difficult to understand what network features learned by SVMs mean biologically. In contrast our method simply counts the PPI between GWAS hits and candidate core genes, and it is easy to know that we are trying to find protein complexes of GWAS hits and core genes.

There are also differences in terms of validation, (Chen et al. 2011) used Crohn's disease GWAS and simulated data, while (Greene et al. 2015) uses hypertension GWAS, OMIM, GO and drug targets from drug bank for validation. In contrast, we applied extensive validation, including overlap with Clinvar pathogenic variants, overlap with drug targets, demonstrated increased disease association in PPI only and PPI-GWAS, compared to GWAS only, and showing that PPI-GWAS tend to have stronger p-values than GWAS only, and demonstrating that core genes containing Clinvar pathogenic variants were enriched for disease relevant pathways. Another novel feature of our paper is that, we applied our method to 1,381 GWAS studies, demonstrating that

our method works across many different diseases and phenotypes. Which is in contrast to many other studies integrating GWAS with PPI which have focused on single diseases such as Multiple sclerosis (Ragnedda et al. 2012)Type 1 diabetes(Bergholdt et al. 2007) obesity (Cheng et al. 2018)and Asthma (Liu et al. 2017).

(Wang et al. 2011) also referenced a study by Oti et. al(Oti et al. 2006) which was perhaps the most similar to ours, however it was published in 2006 and used OMIM genes rather than GWAS hits, with the aim of prioritizing causal genes within disease associated linkage intervals. Our paper is also timely, it is based on the Omnigenic model which was published in 2017, and has already been cited >950 times, while Chen et. al and Wang et. al were published 9 years ago in 2011. In addition, our work opens up new lines of research, because although many groups perform GWAS it is currently not standard to compare GWAS hits with PPI networks to find additional signal.

2) In Figure 3d: the authors show that their core genes are more enriched with constrained genes (according to pLI scores). The authors compare pLI distribution of the core genes vs. all genes in the PPI network. I think the core genes are likley to have higher degress than background, so this analysis should control for degree distribution of two gene sets being compared. Otherwise, the results would show "highly connected" genes are more likely to have high pLI scores. This would be similar to earlier studies showing hub genes are under stronger evolutionary constraint, but this would have no implications on genetics of diseases.

Below is **Figure 3d** from our original submission

d **pLI distribution across core genes**

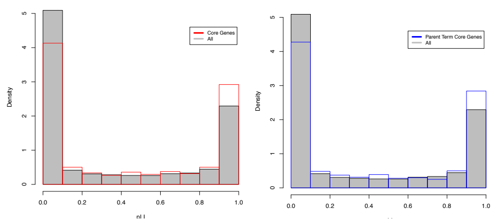

We repeated the pLI analysis but this time we binned the nodes by degree ranging from (1-50, 50-100, 101-150, 150-200, >201), and after binning we did not find any strong enrichment in pLi across candidate core genes (see below), and as a result have removed this analysis from the manuscript.

pLI Individual GWAS studies

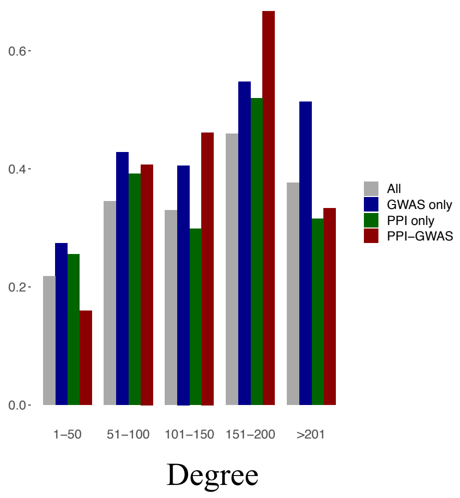

pLI Parent Terms

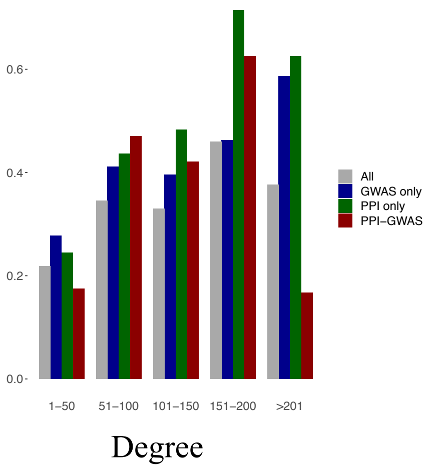

3) The results of cancer core gene analysis are interesting. However, the analysis seems somewhat superficial. I would recommend the authors to perform some deeper analysis. For example, they reported >100 new genes, what are the overepresented biological pathways other than immune genes? Any specific new genes that may provide some new insights on cancer? If one compares across cancer types, are the genes usually found in one cancer type or multiple cancer types?

For example, they reported >100 new genes, what are the overepresented biological pathways other than immune genes?

We took the 78 PPI only cancer parent term candidate core and compared them to the 11,049 genes from the network to assess pathway enrichment .

The top 10 pathways are shown below:

Rank	P-value	Pathway
1	9.62920147510178e-23	GO_STRAND_DISPLACEMENT
2	1.83862171576364e-18	PID_ATM_PATHWAY
3	2.78836265738265e-18	GO_CHROMOSOME
4	2.50837665108936e-17	GO_DOUBLE_STRAND_BREAK_REPAIR

5	3.14648429712284e-15	GO_CELLULAR_RESPONSE_TO_DNA_DAMAGE_STIMULUS
6	3.72009149400043e-15	GO_NUCLEAR_CHROMOSOME
7	4.63937293419238e-15	GO_DNA_SYNTHESIS_INVOLVED_IN_DNA_REPAIR
8	3.97684649599255e-14	GO_DNA_REPAIR
9	3.23696304928964e-13	GO_RESPONSE_TO_IONIZING_RADIATION
10	9.31662085424131e-13	GO_DNA_RECOMBINATION

We found pathways mainly related to DNA repair.

Any specific new genes that may provide some new insights on cancer?

We merged all our cancer germline and somatic candidate core genes, and then removed from this any known cancer genes (cancer GWAS hits, 218 somatically mutated genes, genes from ‘KEGG pathways in cancer’ genes from the cosmic cancer gene census(Tate et al. 2019), known cancer predisposition genes(Rahman 2014) and Oncokb(Chakravarty et al. 2017) cancer drug targets).

After removing known cancer genes we were left with 817 genes, we then split these 817 genes into germline(n=110), somatic (n=633), both germline and somatic (n=74). Then we looked for any pathways that are enriched. Below in red are the top 10 pathways enriched in the germline genes, and in yellow are the top 10 pathways enriched in the somatic genes. It is clear that pathways related to DNA repair are enriched in germline while pathways related to transcription and phosphorylation are enriched in somatic.

GERMLINE

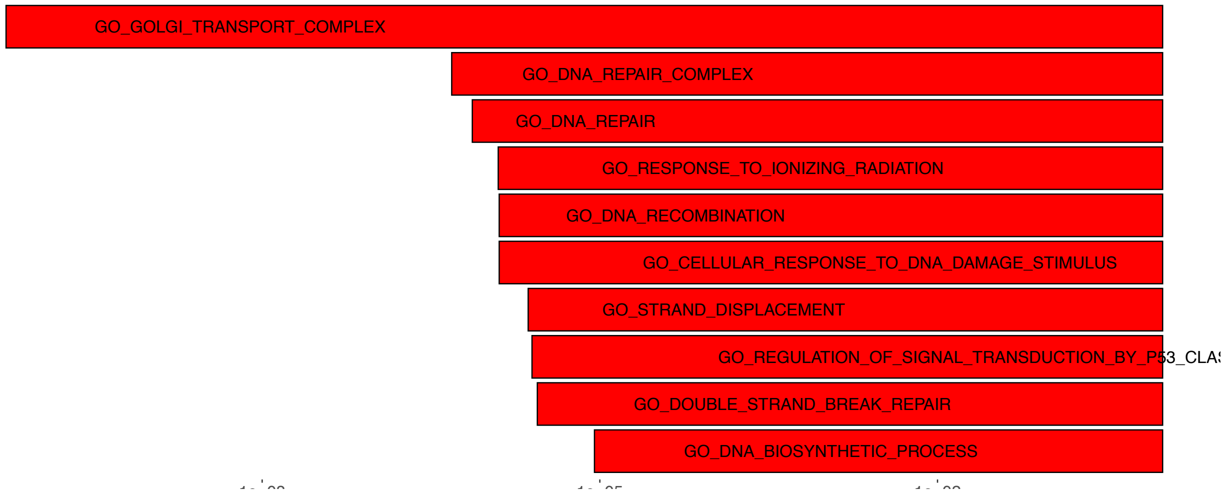

SOMATIC

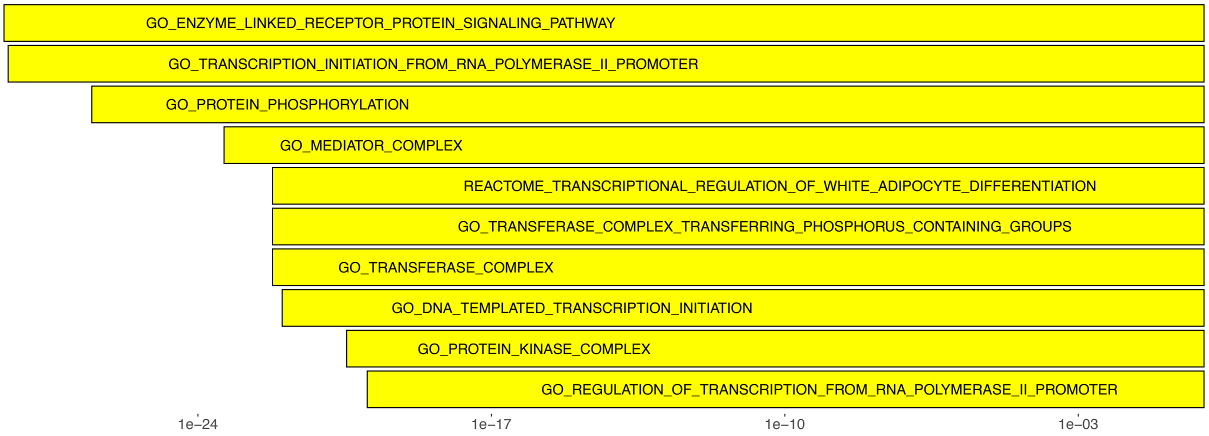

If one compares across cancer types, are the genes usually found in one cancer type or multiple cancer types?

In order to determine whether the cancer candidate core genes tend to be found in one cancer type or more cancer types, we looked at all of 343 individual GWAS studies we detected candidate core genes in and then only kept the core genes from the GWAS studies annotated to the cancer parent term (39 studies). Each of these studies is annotated to an EFO term (disease Ontology term provided by the GWAS catalog). We counted the number of EFO terms, associated with each candidate core gene from the 39 Cancer GWAS studies. Below we have shown the candidate core genes found in 3 or more EFO terms. Which include the candidate core genes *ABL1* and *ESR1*, perhaps not surprisingly we found *TP53* to be a candidate core gene in 5 EFO terms.

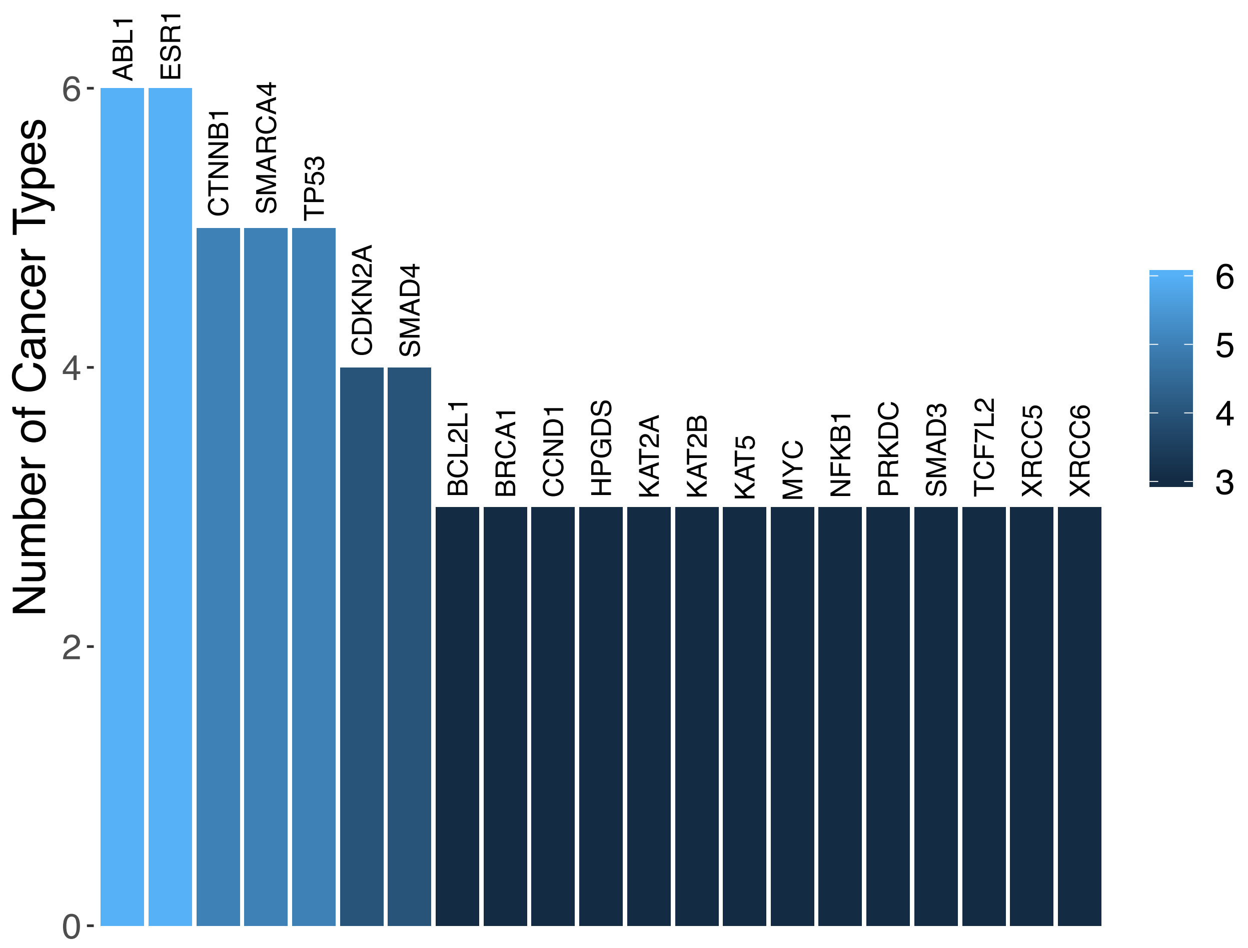

Below are the list of EFO terms corresponding to the candidate core genes, *ESR1 ABL1* and *TP53*

ESR1 endometrial endometrioid carcinoma
breast carcinoma
diffuse large b-cell lymphoma
central nervous system cancer
basal cell carcinoma,
endometrial neoplasm

ABL1 central nervous system cancer
basal cell carcinoma,
carrier status
breast carcinoma
estrogen receptor negative breast cancer
melanoma

TP53	endometrial neoplasm<br>breast carcinoma<br>estrogen receptor negative breast cancer<br>endometrial endometrioid carcinoma<br>neoplasm of mature b-cells

Minor comments

1) Figure legends are generally too long and sometimes redundant with the main text.

We have fixed this

2) Lines 336-340: the authors mentioned that lipid traits have the highest proportion of core genes that are also top GWAS hits, and offered the possible explanation that lipid traits may be less polygenic. While this may well be true, I think it is important to consider the power of studies. For studies with large sample sizes (likely the case for lipid traits), it is likely that many "core" genes would be found as GWAS hits, so the proportion would be high.

We have removed lines suggesting that Lipid traits are less polygenic (**lines 396-398**).

3) Line 358: should cite Figure 3e instead of 3c.

We have fixed this.

References

Bergholdt R, Storling ZM, Lage K, Karlberg EO, Olason PI, Aalund M, Nerup J, Brunak S, Workman CT, Pociot F. 2007. Integrative analysis for finding genes and networks involved in diabetes and other complex diseases. *Genome Biol* **8**: R253.

Boyle EA, Li YI, Pritchard JK. 2017. An Expanded View of Complex Traits: From Polygenic to Omnigenic. *Cell* **169**: 1177-1186.

Burke AC, Dron JS, Hegele RA, Huff MW. 2017. PCSK9: Regulation and Target for Drug Development for Dyslipidemia. *Annu Rev Pharmacol Toxicol* **57**: 223-244.

Calabrese GM, Mesner LD, Stains JP, Tommasini SM, Horowitz MC, Rosen CJ, Farber CR. 2017. Integrating GWAS and Co-expression Network Data Identifies Bone Mineral Density Genes SPTBN1 and MARK3 and an Osteoblast Functional Module. *Cell Syst* **4**: 46-59 e44.

Chakravarty D, Gao J, Phillips SM, Kundra R, Zhang H, Wang J, Rudolph JE, Yaeger R, Soumerai T, Nissan MH et al. 2017. OncoKB: A Precision Oncology Knowledge Base. *JCO Precis Oncol* **2017**.

Chartier-Harlin MC, Crawford F, Houlden H, Warren A, Hughes D, Fidani L, Goate A, Rossor M, Roques P, Hardy J et al. 1991. Early-onset Alzheimer's disease caused by mutations at codon 717 of the beta-amyloid precursor protein gene. *Nature* **353**: 844-846.

Chen M, Cho J, Zhao H. 2011. Incorporating biological pathways via a Markov random field model in genome-wide association studies. *PLoS Genet* **7**: e1001353.

Cheng M, Mei B, Zhou Q, Zhang M, Huang H, Han L, Huang Q. 2018. Computational analyses of obesity associated loci generated by genome-wide association studies. *PLoS One* **13**: e0199987.

Dron JS, Hegele RA. 2016. Genetics of Lipid and Lipoprotein Disorders and Traits. *Curr Genet Med Rep* **4**: 130-141.

Engelender S, Kaminsky Z, Guo X, Sharp AH, Amaravi RK, Kleiderlein JJ, Margolis RL, Troncoso JC, Lanahan AA, Worley PF et al. 1999. Synphilin-1 associates with alpha-synuclein and promotes the formation of cytosolic inclusions. *Nat Genet* **22**: 110-114.

Engelender S, Wanner T, Kleiderlein JJ, Wakabayashi K, Tsuji S, Takahashi H, Ashworth R, Margolis RL, Ross CA. 2000. Organization of the human synphilin-1 gene, a candidate for Parkinson's disease. *Mamm Genome* **11**: 763-766.

Evans D, Arzer J, Aberle J, Beil FU. 2011. Rare variants in the lipoprotein lipase (LPL) gene are common in hypertriglyceridemia but rare in Type III hyperlipidemia. *Atherosclerosis* **214**: 386-390.

Friedman LS, Ostermeyer EA, Szabo CI, Dowd P, Lynch ED, Rowell SE, King MC. 1994. Confirmation of BRCA1 by analysis of germline mutations linked to breast and ovarian cancer in ten families. *Nat Genet* **8**: 399-404.

Fruchterman TM, Reingold E. 1991. Graph drawing by force-directed placement. *Journal of Software: Practice and Experience* **21**: 1129-1164.

Goate A, Chartier-Harlin MC, Mullan M, Brown J, Crawford F, Fidani L, Giuffra L, Haynes A, Irving N, James L et al. 1991. Segregation of a missense mutation in the amyloid precursor protein gene with familial Alzheimer's disease. *Nature* **349**: 704-706.

Greene CS, Krishnan A, Wong AK, Ricciotti E, Zelaya RA, Himmelstein DS, Zhang R, Hartmann BM, Zaslavsky E, Sealfon SC et al. 2015. Understanding multicellular function and disease with human tissue-specific networks. *Nat Genet* **47**: 569-576.

Hall JM, Lee MK, Newman B, Morrow JE, Anderson LA, Huey B, King MC. 1990. Linkage of early-onset familial breast cancer to chromosome 17q21. *Science* **250**: 1684-1689.

Kuchenbaecker KB, Hopper JL, Barnes DR, Phillips KA, Mooij TM, Roos-Blom MJ, Jervis S, van Leeuwen FE, Milne RL, Andrieu N et al. 2017. Risks of Breast, Ovarian, and Contralateral Breast Cancer for BRCA1 and BRCA2 Mutation Carriers. *JAMA* **317**: 2402-2416.

Landrum MJ, Lee JM, Benson M, Brown G, Chao C, Chitipiralla S, Gu B, Hart J, Hoffman D, Hoover J et al. 2016. ClinVar: public archive of interpretations of clinically relevant variants. *Nucleic Acids Res* **44**: D862-868.

Liberzon A, Birger C, Thorvaldsdottir H, Ghandi M, Mesirov JP, Tamayo P. 2015. The Molecular Signatures Database (MSigDB) hallmark gene set collection. *Cell Syst* **1**: 417-425.

Liu Y, Brossard M, Sarnowski C, Vaysse A, Moffatt M, Margaritte-Jeannin P, Llinares-Lopez F, Dizier MH, Lathrop M, Cookson W et al. 2017. Network-assisted analysis of GWAS data identifies a functionally-relevant gene module for childhood-onset asthma. *Sci Rep* **7**: 938.

Miki Y, Swensen J, Shattuck-Eidens D, Futreal PA, Harshman K, Tavtigian S, Liu Q, Cochran C, Bennett LM, Ding W et al. 1994. A strong candidate for the breast and ovarian cancer susceptibility gene BRCA1. *Science* **266**: 66-71.

Murrell J, Farlow M, Ghetti B, Benson MD. 1991. A mutation in the amyloid precursor protein associated with hereditary Alzheimer's disease. *Science* **254**: 97-99.

Nishi M, Nanjo K. 2011. Insulin gene mutations and diabetes. *J Diabetes Investig* **2**: 92-100.

Oti M, Snel B, Huynen MA, Brunner HG. 2006. Predicting disease genes using protein-protein interactions. *J Med Genet* **43**: 691-698.

Ragnedda G, Disanto G, Giovannoni G, Ebers GC, Sotgiu S, Ramagopalan SV. 2012. Protein-protein interaction analysis highlights additional loci of interest for multiple sclerosis. *PLoS One* **7**: e46730.

Rahman N. 2014. Realizing the promise of cancer predisposition genes. *Nature* **505**: 302-308.

Rossin EJ, Lage K, Raychaudhuri S, Xavier RJ, Tatar D, Benita Y, International Inflammatory Bowel Disease Genetics C, Cotsapas C, Daly MJ. 2011. Proteins encoded in genomic regions associated with immune-mediated disease physically interact and suggest underlying biology. *PLoS Genet* **7**: e1001273.

Tate JG, Bamford S, Jubb HC, Sondka Z, Beare DM, Bindal N, Boutselakis H, Cole CG, Creatore C, Dawson E et al. 2019. COSMIC: the Catalogue Of Somatic Mutations In Cancer. *Nucleic Acids Res* **47**: D941-D947.

Wang X, Gulbahce N, Yu H. 2011. Network-based methods for human disease gene prediction. *Brief Funct Genomics* **10**: 280-293.

Wang Y, Zhang S, Li F, Zhou Y, Zhang Y, Wang Z, Zhang R, Zhu J, Ren Y, Tan Y et al. 2020. Therapeutic target database 2020: enriched resource for facilitating research and early development of targeted therapeutics. *Nucleic Acids Res* **48**: D1031-D1041.
