# Supplementary material for: Protein-Protein interactions uncover candidate ‘core genes’ within omnigenic disease networks
Source: PLoS Genet. 2020 Jul 17;16(7):e1008903. doi: 10.1371/journal.pgen.1008903 (PMC7390454; doi:10.1371/journal.pgen.1008903)
Supplement: Attachment — Submitted filename: Response_to_reviewers_round2.pdf [file pgen.1008903.s031.pdf]

Response to reviewers

We thank the reviewers and editors for their enthusiasm for our manuscript and their constructive criticisms that have helped us improve the quality of our work. Below please find a point-by-point response to reviewer comments. Reviewer remarks are in blue and our responses are in black.

REVIEWER 1

Reviewer #1: The authors have provided a thorough response to my comments and suggestions.

We thank you for your comments and feedback, as they have greatly strengthened this manuscript.

Following the comments of Reviewer 2, I am now curious whether the Clinvar analysis and the drug target analysis were corrected for node degree. Otherwise, I only have presentation-related comments:

Authors Response: We thank the reviewer for this important observation and have now added further analysis to address this. We found that the mean node degree tended to be higher in nodes with Clinvar pathogenic variants, COSMIC, ONCOKB and TTD drug targets compared to the whole network(all 11,049 nodes) so we removed all comparisons between various categories like PPI-GWAS, PPI only and GWAS only and the whole network ('All' comparison).

Clinvar Analysis

In the original Clinvar analysis in both individual GWAS studies and parent terms, we found a higher proportion of Clinvar pathogenic variants in PPI only compared to GWAS only and PPI-GWAS compared to GWAS only (see below).

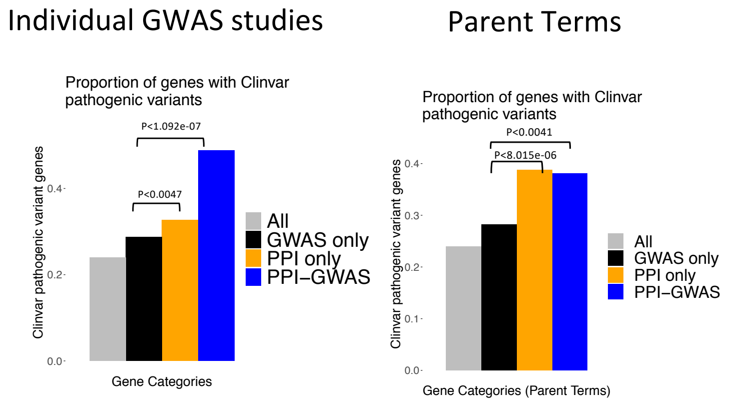

Figure 3c & 4c : (**left**) Proportion of Clinvar pathogenic variant containing genes in All 11,049 nodes, GWAS only, PPI only and PPI-GWAS. We found enrichment in PPI only compared to GWAS only (P<0.0047, Fisher's Exact Test) and in PPI-GWAS compared to GWAS only (P<1.092e-07, Fisher's Exact Test) (**right**) Parent Term Analysis Proportion of Clinvar pathogenic variant containing genes in All 11,049 nodes, GWAS only, PPI only and PPI-GWAS. We found

enrichment in PPI only compared to GWAS only (P<8.015e-06 , Fisher's Exact Test) and in PPI-GWAS compared to GWAS only (P<0.0041, Fisher's Exact Test).

In order to better understand how these patterns might relate to node degree, we stratified the fraction of genes with Clinvar pathogenic variants, into different degree bins, and performed comparisons within each degree bin (see figure below). In general, similar trends were observed in the stratified analysis Supplemental Figure 5 (**S5 Figure)** as the unstratified analysis. As in the unstratified analysis, we observed that both PPI-GWAS and PPI only were more enriched for Clinvar pathogenic variants compared to GWAS only.

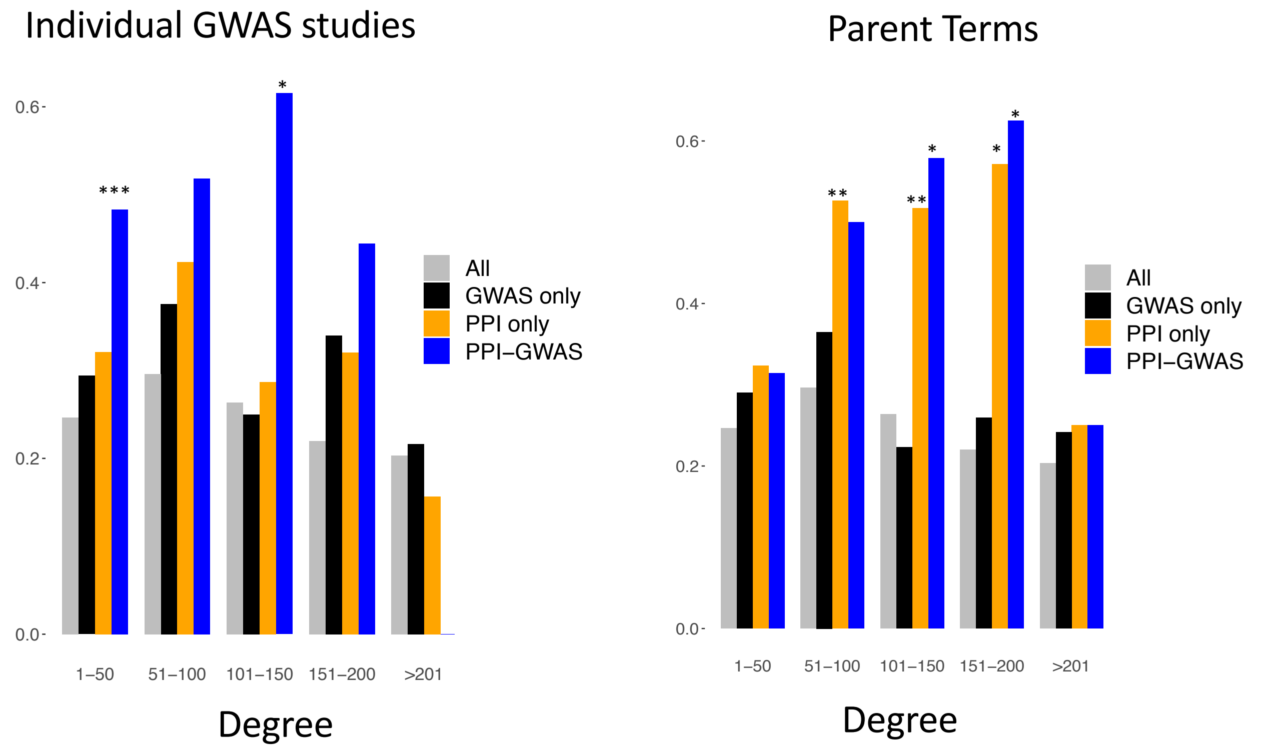

S5 Figure: (left) Individual GWAS studies: after stratifying by degree-bins we found that PPI-GWAS had the highest proportion of Clinvar pathogenic variants across 4 degree bins (statistically significant in 2 bins) consistent with what was observed prior to stratifying by degree**. (right) Parent Term analysis:** we found that PPI only had a greater proportion than GWAS only in 3 bins, and PPI-GWAS has a greater proportion than GWAS only in 2 bins.

COSMIC,ONCOKB and Therapeutic Targets Database (TTD) analysis -GERMLINE

We then performed similar analyses for the drug targets analysis. As a reminder, we display results from the manuscript with unstratified degree below:

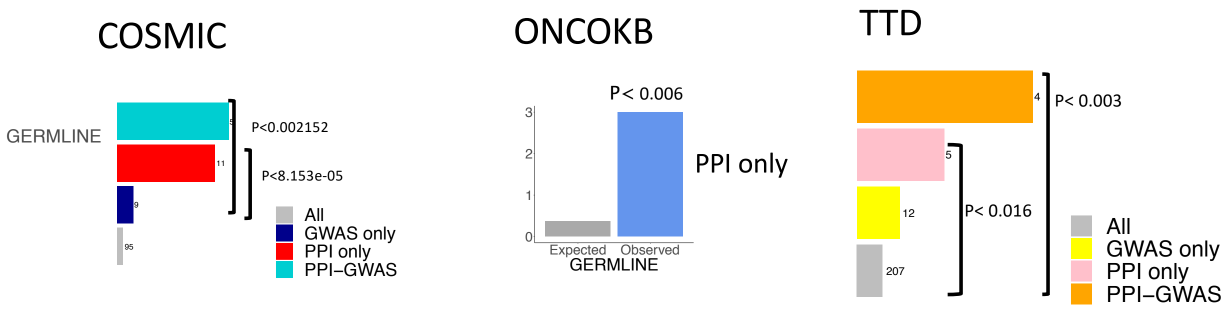

Figures 4d,e,f. Left, COSMIC: Cancer Parent Term candidate core genes were compared to the COSMIC cancer gene census germline genes. We found PPI-GWAS (P<0.002152, Fisher's Exact Test) and PPI-only (P<8.153e-05 , Fisher's Exact Test) were enriched for overlaps compared to GWAS only. The 11 PPI only genes that overlap with COSMIC germline genes are *BLM, SMARCB1, STAT3, NBN, BRCA1, CDH1, SMAD4, AR, WRN, PALB2, CDK4, and the list of* 5 PPI-GWAS that overlap with COSMIC germline genes are, *BRCA2, TP53, ATM, BARD1* and *CDKN2A*. **Middle, ONCOKB:** We obtained cancer drug targets from Oncokb and found enrichment in PPI only(P<0.006) compared to all nodes in the network (n=11,049). The PPI only cancer parent term candidate core genes that are also Oncokb cancer drug targets are (*SMARCB1, CDK4, BRCA1*), while the PPI-GWAS cancer parent term candidate core genes that are also Oncokb cancer drug targets are (ESR1, CDKN2A, BRCA2, ATM). **Right, TTD:** Cancer drug targets were obtained from the Therapeutic Targets Database. We found enrichment in PPI-GWAS (P< 0.002 , Fisher's Exact Test) and PPI only (P< 0.015 , Fisher's Exact Test) compared to all genes in the network. The cancer drug target overlapping PPI only include (*STAT3, PGR, NR3C1, MCL1, AR)* while the cancer drug targets overlapping PPI-GWAS include (*TP53, CCND1, CASP8, BCL2).* Please note in revised manuscript, figure formatting has changed to facilitate visual consistency with stratified analysis.

After stratifying by degree bin we found that many of the patterns observed in the original unstratified analysis remained. In the COSMIC analysis we found that PPI only and PPI-GWAS were enriched compared to GWAS only. In the ONCOKB analysis we found PPI-GWAS were enriched compared to GWAS only and in the TTD analysis we found that PPI-GWAS was enriched compared to GWAS only. We have now added this stratified analysis to the manuscript as Supplemental Figure 7 (**S7 Figure**, see below):

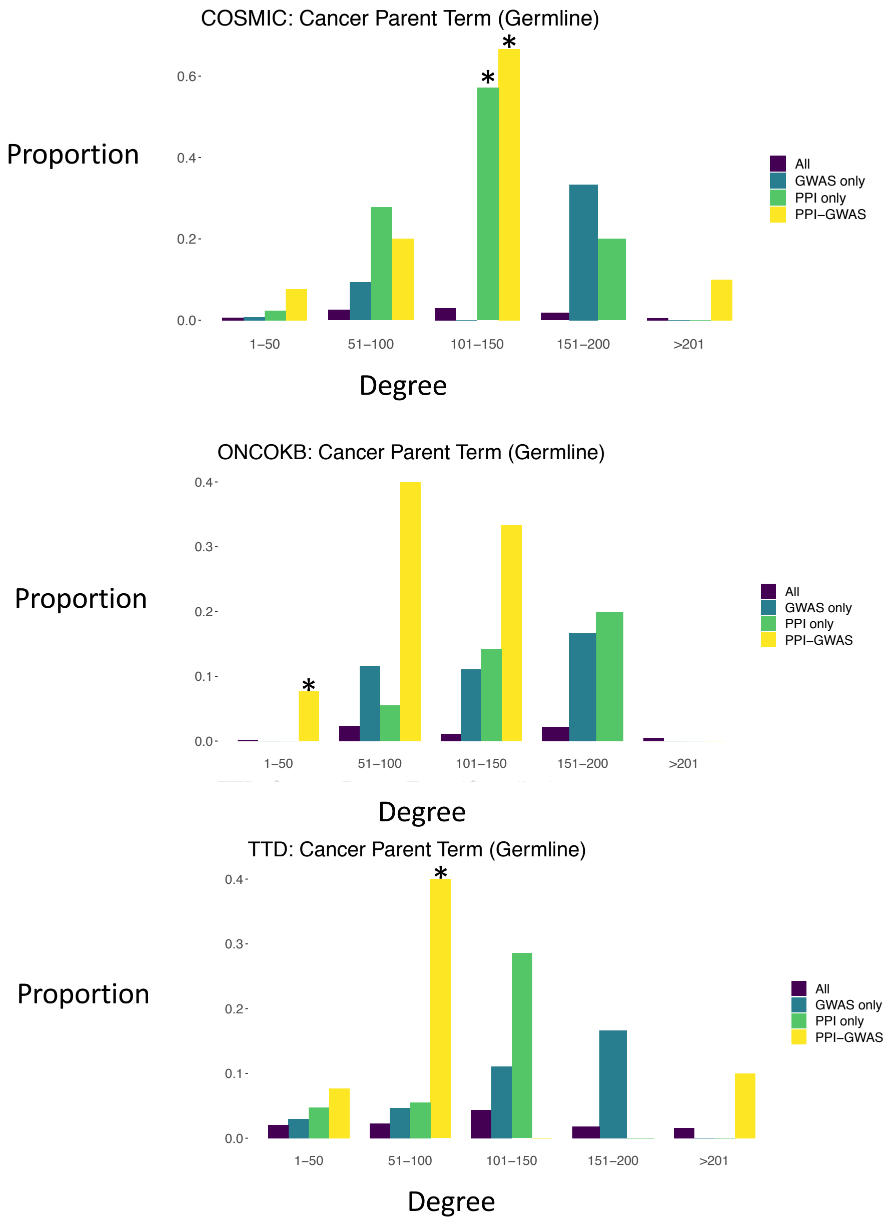

S7 Figure: Similar to the unstratified analysis we found that compared to GWAS only, PPI-GWAS and PPI only were enriched for COSMIC germline variants, and PPI-GWAS was enriched compared to GWAS only in Oncokb variants and PPI-GWAS was enriched compared to GWAS only in cancer drug targets from the Therapeutic Targets Database.

COSMIC,ONCOKB and Therapeutic Targets Database analysis -SOMATIC

As in the cancer germline analysis above, we also performed similar stratified analyses for the cancer somatically mutated analysis, these results are displayed in Supplemental Figure 11 (**S11 Figure**). We have not included those results here for the sake of space. Overall, that analysis revealed that stratifying by degree bin, maintained similar trends as the unstratified analyses. However, we found that the enrichment for cancer drug targets from the Therapeutic Targets Database was less robust after degree stratification, so we have removed that comparison from the main text, but have included the figures in the supplementary Figure 11 (**S11 Figure**) for reference.

• In the current presentation, it feels like you are assuming your hypothesis is true (that ppi genes are candidate core genes) throughout pages 9-15. I would recommend that in this section, you use the term “ppi genes” instead of “candidate core genes”. Then, you could retitle the section starting on page 15 “Clinvar enrichment: ppi genes are candidate core genes.” The title for p. 21 could be “Drug target enrichment: ppi genes are candidate core genes”, and on p. 22, “Cancer driver enrichment: …”.

Thank you for your suggestion, we have renamed ‘candidate core genes’ to ppi genes in these sections and have retitled the various sections as recommended by you. We also included the definition of PPI genes in the schematic on Figure 2a, included a definition of PPI genes in the definition box, and modified the figures to incorporate this terminology.

• The main figures should be readable without referencing the caption. Currently, many axes are unlabeled, and the titles are inconsistent (Figures 3-5). I would recommend adding axis labels everywhere it is not totally obvious and making all titles describe what is being plotted (rather than delivering the punchline).

Thank you for your suggestion, we have remade figures 1b,1c,1d, 2c,3b,3c,3d,3e,4b,c,d and 5b,5d,5e such that they contain axis labels and titles describing what is being plotted.

• Consider including the finding of no excess GWAS signal at ppi-only genes. This analysis suggests that ppi-only genes would not be detected in GWAS of larger sample size.

Thank you for your suggestion we have included this line in the discussion (lines 583-586).

• In many places, a long sentence should be split into two sentences. For example, see lines 85-89, 104-109, 202-205, 250-259, 637-640. In a number of places, a comma should be deleted (e.g. lines 637, 644, 139).

We have fixed these.

REVIEWER 2

Reviewer #2: I congratulate the authors for successfully incorporating reviewer feedback into the manuscript. My concerns with the text have been satisfied, and I have only minor comments below.

We thank the reviewer for their suggestions during the review process of the manuscript and believe their feedback has helped us improve our manuscript.

Line 65: “insulin for” rather than “insulin in”
We have fixed this.

Line 66: BRCA1, as a protein, should not be italicized

We have fixed this.

Line 69: Technically the experiments still have to be performed. It is more accurate to say "by utilizing publicly available reference datasets."
We have changed this line to reflect your suggestion (lines 67-68)

Line 264: delete the second comma
We have fixed this.

Line 386-389: Sentence is difficult to make sense of.
We have fixed this.

Previous version
In fact, analysis of UK biobank data, found protein truncating variants (DeBoever et al. 2018) in *PALB2* associated with breast cancer diagnosis and a family history of breast cancer, and protein truncating variants in *LPL*, associated with decreased risk for high cholesterol(DeBoever et al. 2018).

Current version (lines 354-358)
Consistent with the omnigenic prediction that core genes have deleterious rare variants, is prior analysis of UK biobank data. Which found protein truncating variants (DeBoever et al. 2018) in *PALB2* associated with breast cancer diagnosis and a family history of breast cancer, and protein truncating variants in *LPL* associated with decreased risk for high cholesterol(DeBoever et al. 2018).

Line 473: delete the comma
We have fixed this.

Line 573-586: I think this could be condensed to provide a simple, short summary.
We have fixed this.

Previous version
We demonstrate strong disease relevance amongst our candidate core genes, examples include *BRCA1* in Breast Cancer(Hall et al. 1990; Friedman et al. 1994; Miki et al. 1994; Kuchenbaecker et al. 2017), and *INS* in Type2 diabetes(Nishi and Nanjo 2011). We also demonstrate that GWAS hits that are also candidate core genes (i.e. PPI-GWAS), are more strongly statistically associated with the underlying trait than those loci that are not candidate core genes (i.e. GWAS only). Further, our candidate core genes (PPI-only) are significantly enriched for Clinvar (Landrum et al. 2014) pathogenic variants compared to GWAS only, and previously identified cancer genes obtained from COSMIC(Tate et al. 2019) are preferentially enriched in cancer candidate core genes compared to GWAS only. Finally, using cancer drug targets from the Therapeutics Target Database(Wang et al. 2020) and Oncokb(Chakravarty et al. 2017) we demonstrate that both somatic and germline cancer candidate core genes are enriched for cancer drug targets.

Current version (lines 581-590)
We demonstrate that GWAS hits that are also candidate core genes (i.e. PPI-GWAS), are more strongly statistically associated with the underlying trait than those loci that are not candidate core genes (i.e. GWAS only). In addition, we demonstrate that candidate core genes have no excess GWAS signal (PPI only) - suggesting that they are unlikely to be detected in GWAS of larger sample size. Our results provide unique insights into disease biology and suggest that GWAS can be combined with PPI networks to detect "core genes".

Line 597-603: I think it suffices to say "We present a new application of GWAS data: identifying core genes as envisioned by the omnigenic model that do not themselves exhibit GWAS signal by consulting physical interactions."

We have changed this line to reflect your suggestion (lines 586-588).

Line 629-631: Sentence should be rewritten.

Previous version
Another limitation might be that the Clinvar enrichment at PPI-GWAS compared to GWAS only may be because the GWAS only gene may not be the causal gene at the locus, while PPI-GWAS is.

Current version (lines 627-629)
Another limitation might be that the Clinvar enrichment at PPI-GWAS compared to GWAS only, could be due to the PPI-GWAS gene being the actual causal rather than the GWAS only gene.

Line 637: delete first comma.
We have fixed this

Line 644-653: I recommend deleting this paragraph. It is not terribly helpful.
We have deleted this paragraph.

Line 666-667: As described earlier, the method does not exactly work on every GWAS – the GWAS must have enough hits with PPI edges. It would be better to say that this method adds another layer to interpreting GWAS signal.
We have changed this line to reflect your suggestion (lines 650-651)

Lines 897-906: This should be rewritten so say very clearly that candidate core genes within 1 Mb of a GWAS hit are PPI-GWAS hits while the rest are PPI only. Any GWAS hits without a candidate core gene are GWAS-only.
We have fixed this (lines 878-880).

Lines 949-952: Delete.

We have deleted this.

Lines 1046-1050: Delete.
We have deleted this.

In general: The omnigenic model is by convention not capitalized
We have changed the text so that omnigenic model is no capitalized.

Figure 1c: gene symbol names and axis text too small
Figure 1b step 2: axis text way too small
Figure 3d,e: axis text too small
Figure 4e: axis text too small
Figure 5e: number text too small
Figure 5f: axis text too small
We have fixed this

<u>REVIEWER 3</u>
Reviewer #3: The reviewers have done a good job at addressing my comments. While conceptually related to several papers published in the past, I think the large-scale analysis the authors performed across many GWAS datasets would be a useful resource for the community.

We thank you for your constructive criticism during the review process, comments, and enthusiasm for our manuscript
