# Supplementary material for: Protein-Protein interactions uncover candidate ‘core genes’ within omnigenic disease networks
Source: PLoS Genet. 2020 Jul 17;16(7):e1008903. doi: 10.1371/journal.pgen.1008903 (PMC7390454; doi:10.1371/journal.pgen.1008903)
Supplement: Attachment — Submitted filename: ratnakumar19.docx [file pgen.1008903.s029.docx]

In “Protein-protein interactions uncover ‘core genes’ within Omnigenic disease networks”, Ratnakumar and colleagues investigate pairing protein-protein interaction with genome-wide association study (GWAS) data, as curated by the NHGRI/EBI GWAS Catalog, to nominate ‘core genes’ as conceived by the omnigenic model. The authors hypothesize that core genes will physically interact with GWAS hit genes and develop a graph-based algorithm to call genes that interact with GWAS hits more than expected by chance. They apply this both within individual GWAS and across GWAS annotated with the same GWAS catalog-provided ‘parent term’. Significantly interacting genes (which may contain GWAS hit genes) exhibit stronger signals for loss of function intolerance (pLI scores) and stronger enrichment for curated relevant genes and genes implicated via reportedly pathogenic variants in ClinVAR. This work takes a welcome approach to refining the omnigenic model; however, the current manuscript lacks sufficiently precise methods and language to be assured of its conclusions.

Major Points

1.The distinction between the following terms can get lost: GWAS hits, top GWAS hits, mapped genes, new genes, core genes, interact, etc. Some of this could be clarified in Figure 1 and Figure 2 schematics. It might be easier to follow if you called your candidates as candidate core genes, or hub genes that you later argue serve as core genes. The language interferes with the message. I would argue the phenomena described are more precise than the idea of ‘core genes’, ‘interactions’ and other terms. This concreteness should be embraced rather than obscured.

2.As far as I can tell, many statistical tests are not controlled for node degree. See Figure 3e, lines 431-436, 443, 475-477, 721-727. If I understand the methods, the studies are ascertained to have multiple hits, but I believe this creates a bias in the results in favor of studies that are more likely to have more hit nodes and edges, whereas the all node set will remain at the level of typical connectedness. I recommend subsampling nodes of matched degree AND such that there is at least one edge between them, or removing the requirement altogether. Then determine the significance empirically by creating a null distribution from the samplings. If the significance is driven primarily by differences in degree between GWAS hits and all other genes, that might be a result in its own right, but the interpretation would change.

3. The procedure in 660-681 is hard to follow. I do not understand the two separate steps of Benjamini-Hochberg correction and empirical FDR calculation. It also sounds like the empirical FDR is a p-value, not an FDR. Is it not enough to create one null distribution and then correct for multiple tests on the resulting p-values? This more complicated route, if preferable, requires justification.

4. In lines 333-340 and the legend for Figure 3, you state that the coincidence of core genes and GWAS hits supports the lesser polygenicity of lipid traits. This is repeated in the discussion (line 481). Why this should be the case is unclear to me. There may be a case for polygenicity here but it needs to be fully reasoned to merit inclusion.

5. I am currently not persuaded by the content of most of Figure 4, partially because of the points above. With respect to 4a, I do not understand the significance of immune genes forming a cluster or how that determination was made (the nodes are not marked to distinguish the immune genes). With respect to 4e, it is not enough to say TP53 has more signal than other high degree genes – is this statistically significant? There is an apparent trend (hard to see the way it is plotted) for high degree nodes to have more overlap, so this has to be evaluated more carefully. This section of the manuscript does not concisely communicate the insights made possible by this approach and goes largely unexamined in the Discussion.

6. More broadly, the manuscript is lacking clear proof of usefulness of the work beyond nominating promising genes. I am not convinced this work will be truly impactful without such a demonstration. One possibility is to examine the ‘coreness’ of cancer drug targets or compare this analysis to other published measurements of interest such as heritability (various sources) or polygenicity (O’Connor 2019, AJHG).

7. If the authors are arguing that physical interactions are uniquely positioned to reveal core genes from GWAS hits, it seems that the method should be applied to coexpression or other interaction types for the sake of comparison.

Minor Points

1. I have trouble understanding some parts of the manuscript:

- Line 171
- Line 215-216
- Line 221-223
- Line 253
- Line 588 (By SNP platform do you mean genotyping array?)
- Line 601-606

2. Cut down and clarify some repetitive or long sections, including:

- Line 312-316
- Line 319-320
- Line 401-402
- Line 406-411
- Figure legends in general. Avoid getting into the results in the legend text.

3. There are a number of sentence fragments that can be fixed, including:

- Line 244
- Line 338
- Line 436
- Line 621

4. Text is often too small to read:

- Figure 2 b, d
- Figure 3 b-e
- Figure 4 e

5. Throughout the text, genome-wide association studies are abbreviated as GWAs, but GWAS (with a capital S) is more standard in the field. I advise making this substitution.

6. Other line items:

229-237: The parenthetical remarks are fairly long and do not mix well with references.

248: Remove the double tab

358: you mean 3e, not 3c, I believe.

365/422: Use of ‘new’ to describe interacting genes is confusing. Use ‘tagged’ and ‘untagged’ or ‘tagged’ and ‘interactor’ or something that better captures the class of genes you are describing

406: Fix formatting of “et al.”

572: How are physically interacting genes identified? I am not sure that this is specified in STRING, and if it is, please state which file/column you consulted. I know there is an experimentally determined column; that column encompasses many types of assays, so I advise explaining the scope.

593: I believe the p_value_text column is the p value reported in the text. Thus the filtering step described does not immediately make sense to me. It would be best to elaborate.
